# Dietary supplementation with *Dendrobium officinale* leaves improves growth, antioxidant status, immune function, and gut health in broilers

**DOI:** 10.3389/fmicb.2023.1255894

**Published:** 2023-09-18

**Authors:** Wanqiu Zhao, Yue Chen, Yong Tian, Yunzhu Wang, Jianke Du, Xuan Ye, Lizhi Lu, Chongbo Sun

**Affiliations:** ^1^Institute of Horticulture, Zhejiang Academy of Agriculture Sciences, Hangzhou, China; ^2^State Key Laboratory for Managing Biotic and Chemical Threats to the Quality and Safety of Agro-Products, Institute of Animal Husbandry and Veterinary, Zhejiang Academy of Agriculture Sciences, Hangzhou, China; ^3^Key Laboratory of Livestock and Poultry Resources (Poultry) Evaluation and Utilization, Ministry of Agriculture and Rural Affairs of China, Hangzhou, China; ^4^Zhejiang Xianju Breeding Chicken Farm, Xianju, China

**Keywords:** *Dendrobium officinale* leaves, broiler chicken, immune response, antioxidant capacity, gut microbiota, SCFAs

## Abstract

**Background:**

The *Dendrobium officinale* leaves (DOL) is an underutilized by-product with a large biomass, which have been shown to exhibit immunomodulatory and antioxidant functions. The purpose of this research was to investigate the effects of DOL on broiler growth performance, antioxidant status, immune function, and gut health.

**Methods:**

One hundred and ninety-two 1-day-old chicks were selected and divided into 4 groups at random, 6 replicates for each group and 8 in each. Chicks were given a basal diet supplemented with different amounts of DOL: 0% (control group, NC), 1% (LD), 5% (MD), or 10% (HD). During the feeding trial (70 days), broiler body weight, feed intake, and residual feeding were recorded. On d 70, 12 broilers from each group were sampled for serum antioxidant and immune indexes measurement, intestinal morphological analysis, as well as 16S rRNA sequencing of cecal contents and short-chain fatty acid (SCFA) determination.

**Results:**

In comparison to the NC group, the LD group had greater final body weight and average daily gain, and a lower feed conversion ratio (*p* < 0.05, d 1 to 70). However, in MD group, no significant change of growth performance occurred (*p* > 0.05). Furthermore, DOL supplementation significantly improved the levels of serum total antioxidant capacity, glutathione peroxidase, superoxide dismutase, and catalase, but reduced the level of malondialdehyde (*p* < 0.05). Higher serum immunoglobulin A (IgA) content and lower cytokine interleukin-2 (IL-2) and IL-6 contents were observed in DOL-fed broilers than in control chickens (*p* <0.05). Compared to the NC group, duodenal villus height (VH) and villus height-to-crypt depth (VH:CD) ratio were considerably higher in three DOL supplementation groups (*p* < 0.05). Further, 16S rRNA sequencing analysis revealed that DOL increased the diversity and the relative abundance of cecal bacteria, particularly helpful microbes like *Faecalibacterium*, *Lactobacillus*, and *Oscillospira*, which improved the production of SCFA in cecal content. According to Spearman correlation analysis, the increased butyric acid and acetic acid concentrations were positively related to serum antioxidant enzyme activities (T-AOC and GSH-Px) and immunoglobulin M (IgM) level (*p* < 0.05).

**Conclusion:**

Overall, the current study demonstrated that supplementing the dies with DOL in appropriate doses could enhance growth performance, antioxidant capacity, and immune response, as well as gut health by promoting intestinal integrity and modulating the cecal microbiota in broilers. Our research may serve as a preliminary foundation for the future development and application of DOL as feed additive in broiler chicken diets.

## 1. Introduction

*Dendrobium* is a perennial epiphytic herb belonging to the family Orchidaceae (*Dendrobium* Sw.; Ng et al., 2012), among them, *Dendrobium officinale* kimura et Migo (*D. officinale*) is regarded as one of the most representative *Dendrobium* species in Southeast Asia and China, and has been utilized for over 2,000 years in traditional or folk medicines (Meng et al., 2016). As recorded in the Chinese pharmacopeia (2020 version), stem is the recognized officially medicinal part of *D. officinale* and is usually used for the production of “Fengdou.” Many studies showing that the stem offers a variety of beneficial effects, including inflammation reduction (Li et al., 2020) and intestinal barrier function restoration (Liang et al., 2019). However, a lot of leaves remain on the stems during harvesting, and these are typically thrown without being used, which results in a great waste of resources and exacerbating ecological pressure.

Currently, most researches on *D. officinale* focus on its stems. The stems are known to be abundant in various bioactive components, which include polysaccharides with immunomodulatory activities (Liu et al., 2020), flavonoids with antioxidant effects (Aoi et al., 2021), as well as alkaloids with anti-tumor properties (Bai et al., 2021). The yield and active substances of *D. officinale* leaves (DOL) were reported to be comparable to those of stems, and the fresh leaves even made up nearly 50% of the overall biomass (Zhang et al., 2013). Recently, studies have illustrated that the polysaccharides derived from DOL exhibit regulatory effects on inflammatory cytokines and intestinal microflora, and play a potential therapeutic role in several diseases, including inflammation (Zhong et al., 2022), type II diabetes (Fang et al., 2022), as well as immune deficiency (Xie et al., 2022). Additionally, leaves contain higher amounts of flavonoids than stems, which showed excellent antioxidant activity *in vitro* (Zhou and Lv, 2012; Zhang et al., 2017).

Since the prolonged utilization of antibiotics in food-borne animal husbandry has caused the generation of bacteria with drug resistance that seriously endangered both the ecological environment and human health, antibiotics have now become a critical issue worldwide and are considered one of the greatest hazards to humanity, even though antibiotics enjoyed a golden age from the 1950s to the 1970s (Aminov, 2010). Consequently, the application of antibiotics to promote growth in livestock has been steadily outlawed, encouraging the evaluation of alternative solutions. Thenceforth, researchers have attempted to replace antibiotics with natural bioactive ingredients contained in plants, aiming to improve animal health and growth performance (Lillehoj et al., 2018). For the last few years, a variety of extracts or by-products from plants or traditional Chinese medicine, such as *Acanthopanax senticosus* (Long et al., 2021), ginseng residues (Xiao et al., 2022), *Eucommia ulmoides* (Peng et al., 2022), etc., have been investigated singly or in combination to enhance animal health and productive performance. Moreover, studies have showed that the usage of synthetic molecules was linked to potential hepatotoxicity and carcinogenicity in animals and human, which has boosted consumer demand for natural antibiotics in place of synthetic antibiotics (Mavrommatis et al., 2021).

Considering the content of bioactive substances in DOL and the proven immunostimulatory and antioxidant activities, we expect to transfer this abundant resource into the animal production industry as additives. The object of this experiment was to investigate the effects of adding varying amounts of DOL to the diet on broiler growth performance, immune status, antioxidant capacity, and intestinal flora.

## 2. Materials and methods

### 2.1. Birds, experimental design and diets

We carried out this experiment at Zhejiang Xianju chicken breeding farm (Tiantai, Zhejiang, China) from June to August 2022. Four treatment groups with 6 replicates, each containing 8 chickens, were created at random from 192 1-day-old healthy male broilers with comparable performance. The experiment ran for 10 weeks. After 3 days of keeping the room temperature at approximately 33°C, it was gradually reduced to 24°C by a 1°C reduction every other day and then maintained until the experiment was completed. The experimental birds were given unlimited access to food and water.

Provided broiler chickens with the same basal diet that was formulated in accordance with the nutritional guidelines suggested by the Agricultural Industry Standards of the People’s Republic of China (NY/T 3645-2020; nutrient requirements of yellow chickens). The ingredients, calculated and analyzed nutrient components of basal diets are showed in Table 1.

**Table 1:** Composition and nutrition level of basal diets (%, as-fed basis).

Items	Day 1 to 42	Day 43 to 70
Ingredients (%)		
Corn	53.7	35.6
Soybean meal	23	8.2
Extruded soybean	6	2
Rice bran	6.5	6
Soybean oil	0.8	1.4
Corn gluten meal	3	4
Limestone	1.33	1.3
Premix^1^	4	3.2
Fermented soybean meal	1.67	
Wheat grain		18
Rice bran		6
DDGS (corn)^2^		10
Wheat red dog		0.3
Total	100	100
Calculated nutrient components		
Metabolizable energy(Kcal/kg)	2,950	2,997
Crude fat	4.8	5.5
Crude protein	21.1	16.7
Lysine	1.22	0.95
Tryptophan	0.22	0.19
Methionine	0.54	0.40
Threonine	0.85	0.67
Methionine and Cysteine	0.88	0.72
Calcium	0.87	0.70
Total phosphorus	0.63	0.58
Analyzed nutrient components		
Crude protein	21.12	16.34
Crude fat	4.89	5.58
Crude ash	5.04	5.53
Dry matter	89.75	90.24

^1^Premix provided per kilogram of diet: vitamin A, 1,000 IU; vitamin D_3_, 250 IU; vitamin E (DL-α-tocopheryl acetate), 15 mg; vitamin B_1_, 3.6 mg; vitamin B_2_, 2.8 mg; vitamin B_6_, 4.1 mg; Cu (as CuSO_4_·5H_2_O), 7.5 mg; Fe (as FeSO_4_·7H_2_O), 75 mg; Zn (as ZnSO_4_), 51.75 mg; Mn (as MnSO_4_), 55.65 mg; I (as Ca(IO_3_)_2_), 0.1 mg; Se (as NaSeO_3_·5H_2_O), 0.05 mg. ^2^DDGS, Distillers dried grains with solubles.

The experiment birds were divided into four groups: control group without any addition (NC), and three experimental groups fed a basal diet supplemented with 1%(LD), 5% (MD), and 10% (HD) of *Dendrobium Officinale* leaf (DOL) powder, respectively. The DOL was offered by Zhejiang Tiefengtang Bio-technology Co., Ltd. (Wenzhou, Zhejiang, China) and previously powdered. The feeds were placed in a dry and well-ventilated storehouse before usage, packaged in sealed plastic bags.

### 2.2. Chemical analysis

The contents of dry matter, ether extract, crude protein, and crude ash in the basal diets and dried powder of DOL were measured according to standard procedures of the Association of Official Analytical Chemists (AOAC; International, 2007). Crude fiber content in DOL was determined in accordance with the method in GB/T 5009.10-2013 (in Chinese). Furthermore, we also measured the contents of main bioactive compounds in DOL, including polysaccharides, flavonoids, polyphenols, and alkaloids (Table 2). Analysis was performed in three duplicates.

**Table 2:** The main bioactive compounds and chemical composition in dried powder of *Dendrobium officinale* leaf.

Bioactive compound, mg/g		Chemical composition, g/kg	
Total polysaccharides	85.0	Ether extract	42.01
Total flavonoid	4.57	Crude protein	93.12
Total polyphenol	4.45	Crude ash	109.15
Alkaloids	23.25	Crude fiber	104.00

### 2.3. Growth performance

On days 1, 21, 42, and 70 of the experiment, recorded broiler body weight (BW), feed intake, and residual feeding and calculated the average daily feed intake (ADFI) and average daily gain (ADG) values. The ADFI and ADG were used to calculate the feed-to-gain ratio (F/G) for each trial phase.

### 2.4. Sample collection

One 12-h-fasted broiler was chosen at random from each cage (*N* = 6 per group) on day 42. A 2-mL blood sample was obtained from wing vein and centrifuged at 3,000 × g for 15 min at 4°C, collected serum samples and kept at −20°C for subsequent investigation.

On day 70, 12 broilers per group were randomly chosen for sampling. A blood sample of 5 mL was obtained from the jugular vein of each broiler, and then centrifuged at 3,000 × g for 15 min at 4°C, in order to collect the serum samples, which was then kept at −20°C until the immune and antioxidant analyses. The chickens were subsequently euthanized by exsanguination, and their internal immune organs, including the thymus, liver, spleen, and bursa of Fabricius, were collected and weighted individually. Eviscerated yield was defined as the weight after removal of feathers, head, feet, abdominal fat, as well as all viscera. The yields of the breast and leg muscle were determined by calculating the ratio of breast or leg muscle weight to the eviscerated weight.

Within 5–10 min after their sacrifice, the cecal were removed aseptically, and the fresh content samples (*N* = 12 per group) were collected into 2-mL sterilized tubes, and immediately froze the tubes in liquid nitrogen, then kept at −80°C for the investigation of intestinal microbiota and short chain fatty acid (SCFA) contents. The small intestinal tissues (duodenum, jejunum, and ileum) were separated, and samples of approximately 4 cm long were taken from the mid-section of each segment. These samples were then fixed with 4% formaldehyde for 15 min and preserved at room temperature for morphological analysis. The remaining segments were split longitudinally, rinsed with a solution of normal saline that had been previously cooled, and then placed in cryotubes. These tubes were frozen instantly in liquid nitrogen and stored at -80°C before gene expression analysis.

### 2.5. Serum immune and antioxidant indexes measurement

The concentrations of immunoglobulin A (IgA), IgG, and IgM, as well as cytokines interleukin 2 (IL-2) and IL-6 in serum samples were detected following the instructions using commercial chicken-specific ELISA kits (Beijing Huaying Biotechnology Institute, Beijing, China) to assess the status of immune response.

Clinical chemistry assay kits (Beijing Huaying Biotechnology Institute, Beijing, China) were used to measure the superoxide dismutase (SOD), catalase (CAT), total antioxidant capacity (T-AOC), glutathione peroxidase (GSH-Px), and malondialdehyde (MDA) levels in serum samples, in accordance with the manuals.

### 2.6. Morphological analysis of small intestinal tissues

The duodenum, jejunum, and ileum segments were embedded in paraffin wax, sectioned (3 μm) and stained with hematoxylin and eosin. The distance between the tip of villus and the top of the laminate propria was defined as the villus height (VH), and the distance between the villus-crypt axis and the tip of the muscular mucosa was recognized as crypt depth (CD). Utilizing the Image-Pro Plus 6.0 (Media Cybemetics, Inc., MD, United States) in combination with light microscopy (Eclipse Ci-L, Nikon, Japan), three VH and CD values were determined for each intestinal sample, and the VH-to-CD ratio was then calculated.

### 2.7. RNA extraction and quantitative real-time PCR

Followed the manufacturer’s instruction of RNAsimple Total RNA Kit (Tiangen Biotech, Co. Ltd., Beijing, China), we extracted the total RNA of small intestinal tissue samples, which were measured the concentrations spectrophotometrically and uniformly diluted to 1,000 ng/μL. Subsequently, after the removing of contaminated genomic DNA, the reverse transcription of 1 μg RNA was conducted for each sample (*N* = 3 per group) using FastKing RT Kit (With gDNase; Tiangen Biotech, Co. Ltd., Beijing, China). The total volume of quantitative real-time polymerase chain reaction (RT-qPCR) reaction mixture was 10 μL, containing 5 μL of TB Green® Premix Ex Taq™ II (Tli RNAseH Plus; TaKaRa Biotechnology Inc., Japan), 2.2 μL of ddH_2_O, 2 μL of template cDNA, and 0.4 μL of each forward and reverse primer. We performed qRT-PCR reactions on a LightCycler96 real-time PCR system (Roche Applied Science, Indianapolis, IN, United States) with the following amplification procedure: 95°C for 30 s, 95°C for 5 s, and 60°C for 20 s. Steps 2–3 were repeated for 40 cycles. There were three duplicates of each reaction. The gene expression level in the same group was normalized to that of the housekeeping gene—β-actin, and 2^−ΔΔCt^ method was applied to calculated the relative expression of mRNA (Livak and Schmittgen, 2001). We utilized Primer Premier 5.0 software^1^ to design the qRT-PCR primers for this experiment based on the genes of *Gallus gallus*, and synthesized them at Generay Biotechnology Co., Ltd. (Shanghai, China). Table 3 presents the primer information.

**Table 3:** The RT-PCR primer sequence information.

Gene name	Forward primer (5′-3′)	Reverse primer (5′-3′)	PCR product (bp)
*β-actin*	CTGGCACCTAGCACAATGAA	ACATCTGCTGGAAGGTGGAC	109
*ZO-1*	CTTCAGGTGTTTCTCTTCCTCCTC	CTGTGGTTTCATGGCTGGATC	131
*ZO-2*	CTCCGTCAGCAGGGAACAA	TTGGGCGTGACGTATAGCTG	80
*OCLN*	GTCTGTGGGTTCCTCATCGT	GTTCTTCACCCACTCCTCCA	156
*CLDN1*	CATACTCCTGGGTCTGGTTGGT	GACAGCCATCCGCATCTTCT	100
*CLDN2*	CTGCTCACCCTCATTGGA	AACTCACTCTTGGGCTTCTG	140
*CLDN3*	CTTCATCGGCAACAACATCGTGAC	CCAGCATGGAGTCGTACACCTTG	131
*SOD1*	ACCAAAAGATGCAGATAGGCAC	GGTACGGCCAATGATGCAGT	127
*SOD2*	GGAGGGGAGCCTAAAGGAGAAT	CCCAGCAATGGAATGAGACCT	215
*CAT*	CGCATGTCCGTTTCAGGAGA	AGATAGAAGTCTCGCACCTGAG	80
*GPx*	AATTCGGGCACCAGGAGAA	TCACCTCGCACTTCTCGAAC	114
*Nrf2*	GATAGTGACCCAGTCTTCATTTC	CTTGGTTTCAGGGCTCGT	201

*CAT*, catalase; *CLDN*, Claudin; *GPx*, glutathione peroxidase; *Nrf2*, Nuclear factor erythroid 2-related factor 2; *OCLN*, Occludin; *SOD*, superoxide dismutase; *ZO*, Zonula occludens.

### 2.8. Microbiota profiling by 16S rRNA sequencing

#### 2.8.1. Microbial genomic DNA extraction

The cecum contents of 12 chickens per group were taken to extract total microbial DNA via the OMEGA Soil DNA kit (OMEGA Bio-Tek, Norcross, GA, United States), as directed by the manual. Thermo Fisher Scientific NanoDrop NC2000 spectrophotometer and agarose gel electrophoresis were employed, respectively, to evaluate the quantity and quality of the isolated DNA, which was then kept at −20°C for subsequent analysis.

#### 2.8.2. PCR amplification and sequencing

PCR amplification of the V3–V4 region of the bacterial 16S rRNA gene was conducted using the universal primers: the forward (338F 5′-ACTCCTACGGGAGGCAGCA-3′) and the reverse (806R 5′-GGACTACHVGGGTWTCTAAT-3′). The PCR procedures employed here contained an initial denaturation (98°C for 5 min), followed by 25 cycles consisting of denaturation (98°C for 30 s), annealing (53°C for 30 s), and extension (72°C for 45 s), with a final extension at 72°C for 5 min. Subsequently, performed the purification and quantification of PCR amplicons using Vazyme VAHTSTM DNA Clean Beads (Vazyme, Nanjing, China) and Quant-iT PicoGreen dsDNA Assay Kit (Invitrogen, Carlsbad, CA, United States), respectively. Following the separate quantification step, these amplicons were pooled in equal amounts and sequenced on the Illumina NovaSeq platform for paired end reads of 250 base pairs (bp) with NovaSeq 6000 SP Reagent Kit (500 cycles), which were conducted at Shanghai Personal Biotechnology Co., Ltd. (Shanghai, China).

#### 2.8.3. Bioinformatics analysis of sequencing data

The QIIME2 2019.4 (Bolyen et al., 2019) was applied to conduct the microbiome bioinformatics analyses after slight modifications and refinements to the process according to the official tutorials.^2^ In brief, following the demultiplexing of the raw sequence data with the demux plugin, primer excision was carried out with the cutadapt plugin (Martin, 2011). Then, DATA2 plugin (Callahan et al., 2016) was utilized to quality filter, denoise, merge, and remove chimera from the sequences. To obtain abundance data, non-singleton amplicon sequence variations (ASVs) were matched with Mafft (Katoh et al., 2002). ASVs taxonomic classification was achieved by the classify-sklearn naïve Bayes taxonomy classifier in feature-classifier plugin (Bokulich et al., 2018) against the SILVA Release 132 Database (Koljalg et al., 2013).

Sequence data analyses were mostly processed using QIIME2 and R package (v3.2.0). Alpha diversity (α-diversity) indexes, including Chao1 richness estimator, Shannon diversity index, Simpson index, Pielou’s evenness, observed species, and Good’s coverage, were calculated in QIIME2 and displayed as box plots to reflect the richness and uniformity of cecal microbial communities. The structural variation of microbial communities among samples was investigated by beta diversity (β-diversity) analysis, which was carried out using UniFrac distance metrics (Lozupone and Knight, 2005; Lozupone et al., 2007), and the results were illustrated via principal coordinate analysis (PCoA; Ramette, 2007). Applying QIIME2, using Permutational multivariate analysis of variance (PERMANOVA; McArdle and Anderson, 2001) to examine the significance of variations in microbiota structure across groups. Moreover, to further differentiate the bacteria between all of the groups, a combination of the linear discriminate analysis (LDA) with a score > 2.5 and the effect size measurements (LEfSe) was employed in this study.

All raw sequences were submitted to the National Center for Biotechnology Information (https://www.ncbi.nlm.nih.gov/sra, NCBI) Sequence Read Archive with the deposit number of BioProject PRJNA942368.

### 2.9. Target metabolites analysis of SCFAs

According to the protocol for SCFAs analysis in a study previously reported (Zhang S. et al., 2019), the gas chromatography–mass spectrometry (GC-MS) detection method was utilized to measure the concentrations of SCFAs in four cecal content samples per group. Briefly, we firstly prepared the samples as follows: (i) samples were homogenized in 500 μL water with 100 mg glass beads for 1 min; (ii) after centrifugation at 12,000 rpm for 10 min at 4°C, collected 200 μL supernatant and added 100 μL 15% phosphoric acid, 20 μL internal standard solution (375 μg/mL 4-methylvaleric acid), and 280 μL ether, then conducted vortex for 1 min; (iii) the samples were subsequently centrifuged at 4°C for 10 min at 12,000 rpm, and the separated supernatant was transferred into the vial for GC-MS analysis. Sigma-Aldrich (Shanghai, China) supplied the SCFA external standards (acetic acid, butyric acid, isobutyric acid, propionic acid, isovaleric acid, and valeric acid), as well as internal standard (4-methylvaleric acid). Later, the GC analysis was conducted on trace 1,300 gas chromatograph (Thermo Fisher Scientific, United States) with an Agilent HP-INNOWAX (30 m × 0.25 mm ID × 0.25 μm) capillary column, and the carrier gas was helium at 1.0 mL/min. A 10:1 ratio, a 1 μL injection volume, and 250°C for injection port temperature were set for the split mode injection. The ion source and MS transfer line had respective temperatures of 300°C and 250°C. The chromatographic column heating program was as follows: (i) set the initial temperature oven 90°C; (ii) increase the temperature to 120°C at a speed of 10°C/min; (iii) increase the temperature to 150°C with 5°C/min; (iv) increase the temperature to 250°C with 25°C/min, and maintain for 2 min. The ISQ 7000 mass spectrometer (Thermo Fisher Scientific, United States) was applied to conduct mass spectrometric detection in electron bombardment ionization (EI) source mode. With an electron energy of 70 eV, single ion monitoring (SIM) was used as the scanning mode.

Finally, injected the standard and samples into the GC-MS system, and the above program was run to create the unique calibration curves for each external standard by calculating the ratio of their peak area to that of the internal standard. Concentrations of the corresponding metabolites in samples were then calculated according to their peak area ratio to that of the internal standard.

### 2.10. Statistics analysis

The statistical analysis of data was conducted with one-way analysis of variance (ANOVA) using SPSS statistical software (v.22, IBM Corp., NY, United States). The difference significance between the means of experimental groups was determined with Scheffe test at 5% probability level. These results were visualized in GraphPad Prism 7.0 (GraphPad Software Inc., CA, United States). Variation in the data was expressed as pooled standard error of means (SEM) and the significance level was set at *p* < 0.05. Spearman correlation analysis was performed, and a heat map was generated in Origin 2021 (OriginLab, United States).

## 3. Results

### 3.1. Growth performance

Table 4 illustrates the influence of dietary supplementation with DOL on the growth performance of yellow-feathered broilers. The findings showed that 1% DOL supplementation significantly improved the ADGs during d 1–21, d 43–70, and d 1–70, and the final body weight of broilers (*p* < 0.05), but significantly decreased the ADFI during d 1–21, as well as the F/G in the periods of d 1–21 and d 1–70 (*p* < 0.05). No significant difference (*p* > 0.05) between the growth performance of chickens in 5% DOL-fed group and the control group. However, supplemented with 10% DOL showed significantly lower final body weight and ADGs during d 22–42 and d 1–70 than those in the NC group (*p* < 0.05).

**Table 4:** The effects of DOL supplementation on broiler growth performance.

Items	NC	LD	MD	HD	SEM	*p*-value
Initial BW (g)	32.33	32.93	32.99	32.07	0.256	0.053
Final BW (g)	1190.15^b^	1257.15^c^	1180.82^b^	1108.01^a^	14.600	<0.001
d 1–21						
ADFI (g)	18.33^b^	17.16^a^	18.00^b^	16.63^a^	0.149	<0.001
ADG (g)	9.90^ab^	10.65^c^	10.34^bc^	9.66^a^	0.142	0.001
F/G	1.85^b^	1.61^a^	1.74^ab^	1.73^ab^	0.032	0.001
d 22–42						
ADFI (g)	40.10^ab^	40.59^b^	39.26^ab^	38.25^a^	0.481	0.015
ADG (g)	16.92^b^	16.83^b^	16.49^b^	15.59^a^	0.203	0.001
F/G	2.37	2.41	2.38	2.45	0.026	0.150
d 43–70						
ADFI (g)	65.76^bc^	66.44^c^	64.38^ab^	63.64^a^	0.418	0.002
ADG (g)	21.43^a^	23.26^b^	21.07^a^	19.69^a^	0.391	<0.001
F/G	3.07^ab^	2.87^a^	3.06^ab^	3.24^b^	0.052	0.005
d 1–70						
ADFI (g)	44.58^b^	44.66^b^	43.66^ab^	42.66^a^	0.244	<0.001
ADG (g)	16.78^b^	17.74^c^	16.64^b^	15.59^a^	0.211	<0.001
F/G	2.66^b^	2.52^a^	2.63^ab^	2.74^b^	0.029	0.001

BW, body weight; ADFI, average daily feed intake; ADG, average daily gain; F/G, feed to gain rate. ^a–c^Means within same row carrying different superscript letters indicate significant differences (*p* < 0.05).

### 3.2. Carcass traits and relative weights of organs

Dietary addition with 1% and 5% did not significantly affect the weights of carcass, liver, thymus, spleen and bursa relative to the live weight (*p* > 0.05), but the spleen index was significantly higher in the HD group compared to the NC group (*p* < 0.05), as shown in Table 5.

**Table 5:** The effects of dietary DOL on carcass traits and relative weights of organs in broilers.

Items	NC	LD	MD	HD	SEM	*p-*value
Carcass traits						
Carcass yield (%)	83.15^ab^	84.66^a^	83.30^ab^	81.18^b^	0.40	0.023
Eviscerated yield (%)	73.20	74.63	73.21	71.56	0.41	0.067
Breast meat (%)	11.10	11.47	11.54	11.40	0.18	0.832
Thigh meat (%)	18.25	18.58	18.88	19.58	0.22	0.187
Relative weights of organs						
Liver (%)	1.68	1.74	1.74	1.81	0.03	0.578
Thymus (%)	0.51	0.58	0.61	0.56	0.03	0.648
Spleen (%)	0.17^b^	0.18^ab^	0.22^ab^	0.25^a^	0.01	0.005
Bursa of Fabricius (%)	0.25	0.25	0.25	0.29	0.02	0.782

The spleen, liver, and bursary of Fabricius were expressed as a percentage of final body weight (g/100 g body weight). ^a,b^Means within same row carrying different superscript letters indicate significant differences (*p* < 0.05).

### 3.3. Intestinal morphology analysis of small intestine

**Figure 1:**
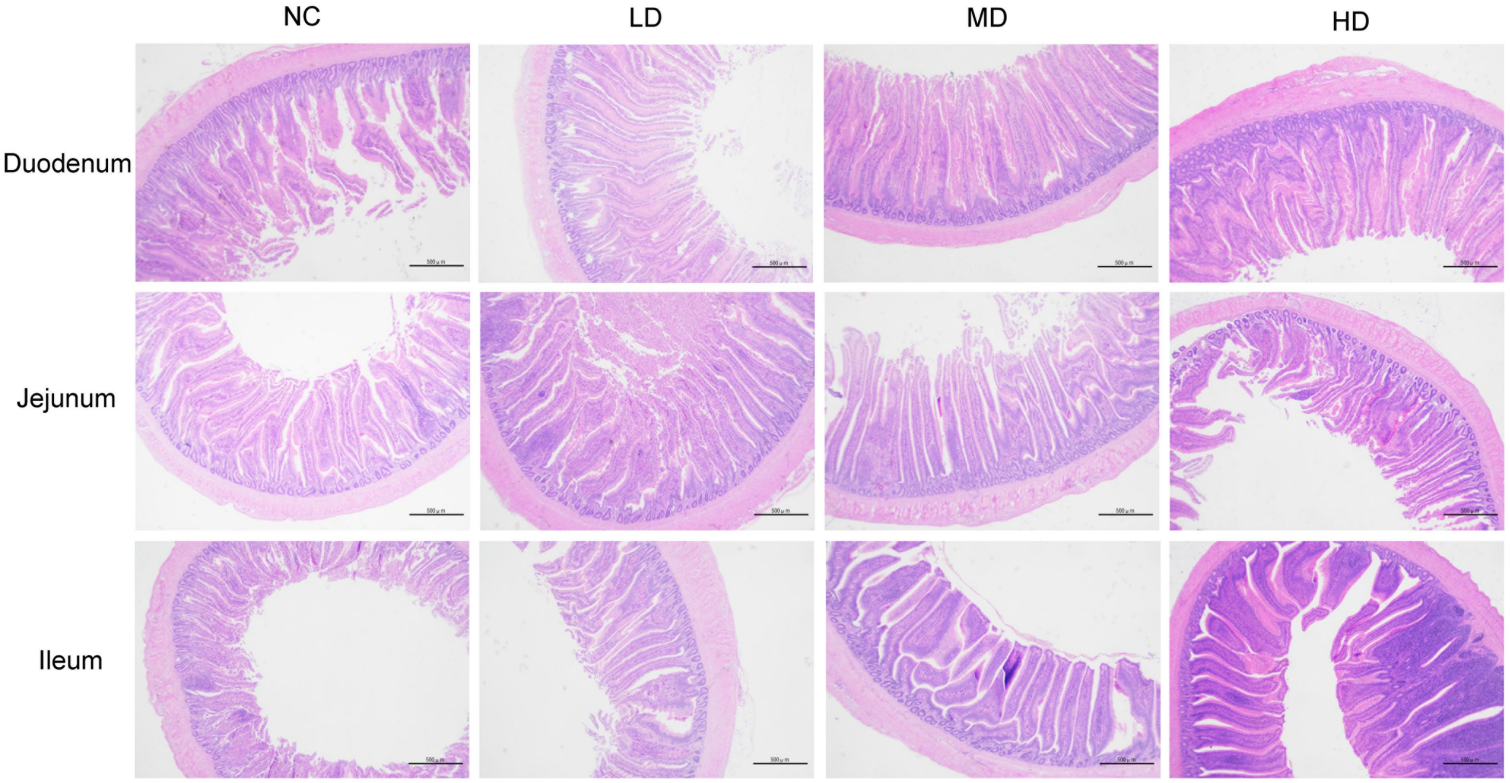
Photomicrograph of the small intestine: hematoxylin and eosin stained duodenum, jejunum, and ileum.

Small intestinal tissues (duodenum, jejunum, and ileum) were stained with hemotoxin and eosin in order to analyze the effects of DOL supplementation on internal morphology (Figure 1 and Table 6). In the duodenum, the significant longer (*p* < 0.05) VH was observed in three DOL supplemental groups compared to the control group. Conversely, the duodenal CD of these groups was all decreased compared with NC group, and the MD and HD showed a significantly decrease (*p* < 0.05). Thus, the VH:CD was significantly improved in three experimental groups (*p* < 0.05). Jejunal VH and VH:CD was significantly higher in MD group than LD group (*p* < 0.05), but the difference between the control group and three experimental groups were not significant (*p* > 0.05). In all three DOL supplementation groups, the ileal VH values were significantly increased (*p* < 0.05) compared to the control group, whereas the ileal CD and VH:CD did not undergo any significant variation (*p* > 0.05).

**Table 6:** The effects of DOL on intestinal villus height and crypt depth.

Items	NC	LD	MD	HD	SEM	*p*-value
Duodenum						
VH, μm	845.49^b^	1146.38^a^	1169.28^a^	1047.53^a^	40.696	<0.001
CD, μm	181.60^a^	161.30^ab^	149.79^b^	119.19^c^	6.982	0.009
VH:CD	4.68^c^	7.13^b^	7.81^ab^	8.79^a^	0.468	<0.001
Jejunum						
VH, μm	934.56^ab^	790.61^b^	1065.99^a^	836.40^ab^	38.620	0.022
CD, μm	141.30	123.43	148.53	121.28	5.268	0.179
VH:CD	6.64^ab^	6.43^b^	7.18^a^	6.89^ab^	0.104	0.031
Ileum						
VH, μm	635.74^b^	860.98^a^	823.35^a^	842.73^a^	32.984	0.021
CD, μm	119.28	134.62	131.08	134.85	3.060	0.242
VH:CD	5.34	6.39	6.28	6.26	0.177	0.103

VH, villus height; CD, crypt depth; VH:CD, villus height to crypt depth ratio. ^a–c^Means within same row carrying different superscript letters indicate significant differences (*p* < 0.05).

### 3.4. Effects of dietary DOL on immune function

**Figure 2:**
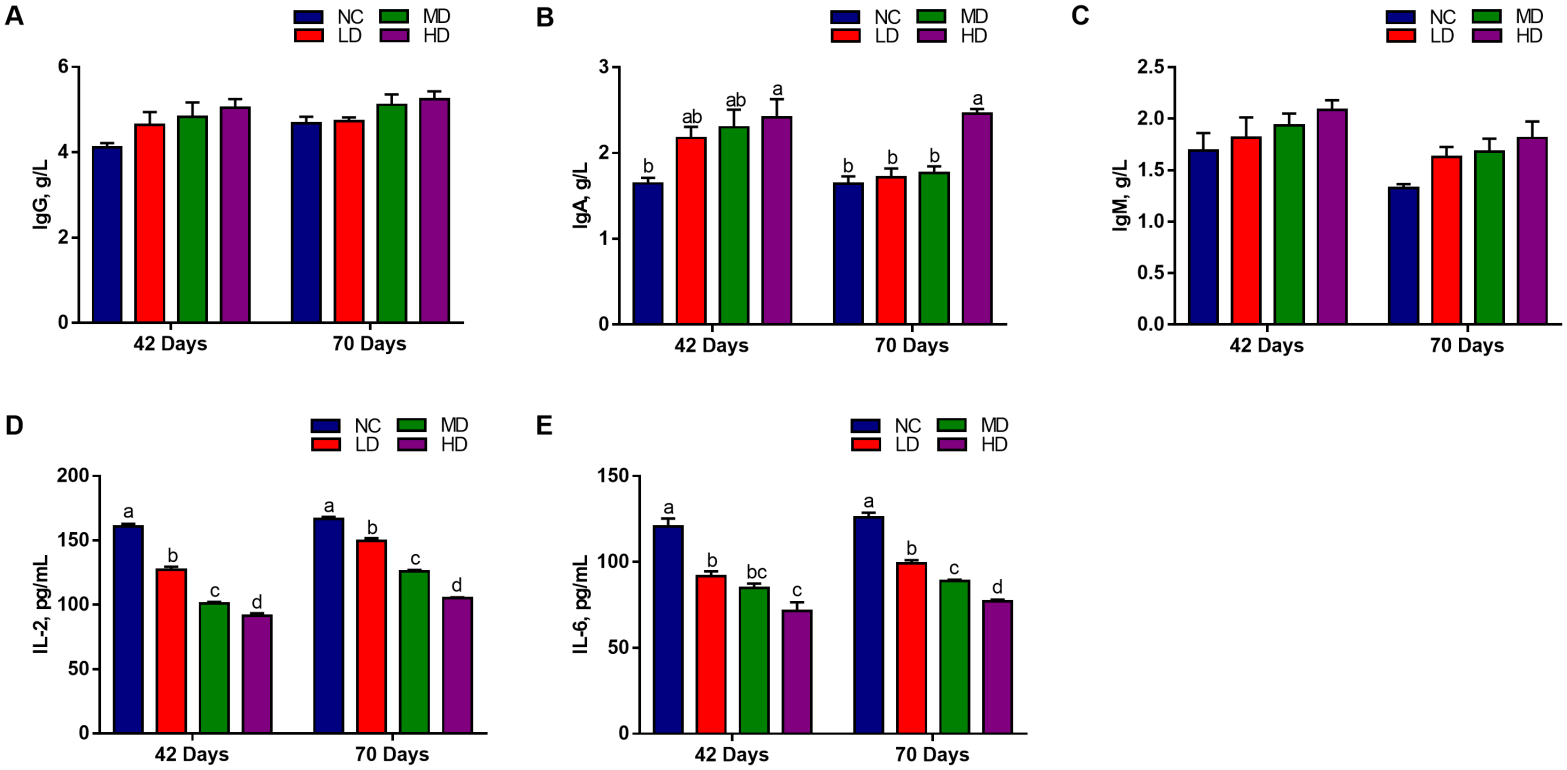
The effects of DOL on the immune indexes and inflammatory factors of broilers. **(A)** IgG, immunoglobulin G; **(B)** IgA, immunoglobulin A; **(C)** IgM, immunoglobulin M; **(D)** IL-2, Interleukin 2; **(E)** IL-6, interleukin 6. The bars represent the mean ± SE. Statistically significant differences between the groups are denoted by different letters over the bars (*p* < 0.05).

We measured the levels of immunoglobulin and cytokines in serum to assess the effects of dietary DOL addition on immune response. Figure 2 shows that the IgG and IgM levels of three DOL supplementation groups were higher at 42 days and 70 days than NC group, but did not reach a significant difference level (*p* > 0.05). The HD group had the highest IgA content, and was significantly different with that in NC group (*p* < 0.05). On the contrary, IL-2 and IL6 contents were significantly decreased (*p* < 0.05) in the three treatments than in the NC group.

### 3.5. Effects of dietary DOL on serum antioxidant capacity

The serum antioxidant profiles of chicken on day 42 and 70 are presented in Table 7. On d 42, significantly elevated (*p* < 0.05) concentrations of serum SOD, T-AOC, CAT, and GSH-Px, and significant lower (*p* < 0.05) MDA level were observed in broilers fed diets supplemented with 5 and 10% DOL compared to those of broilers in control group. No significant variation (*p* > 0.05) was observed in the indexes, except for SOD, between LD and NC. On d 70, the contents of indexes, including SOD, GSH-Px, T-AOC, and CAT significantly increased (*p* < 0.05) in three experimental groups (except for SOD in LD group) than the NC group. On the contrary, there was a significantly lower (*p* < 0.05) concentration of MDA in the serum with the supplementation of DOL.

**Table 7:** The effects of DOL on the serum antioxidant indexes.

Items	NC	LD	MD	HD	SEM	*p* value
d 42						
SOD (U/mL)	45.03^a^	51.07^b^	55.91^b^	65.18^c^	1.336	<0.001
T-AOC (U/mL)	4.97^a^	5.61^ab^	6.34^b^	7.26^c^	0.165	<0.001
GSH-Px (U/mL)	370.71^a^	384.22^a^	414.41^b^	466.73^c^	3.357	<0.001
CAT (U/mL)	24.75^a^	26.69^a^	30.97^b^	36.31^c^	0.869	<0.001
MDA (nmol/mL)	6.34^c^	5.63^bc^	5.18^ab^	4.57^a^	0.182	<0.001
d 70						
SOD (U/mL)	52.70^a^	56.10^a^	63.07^b^	70.62^c^	1.207	<0.001
T-AOC (U/mL)	6.42^a^	7.50^b^	8.13^c^	8.91^d^	0.123	<0.001
GSH-Px (U/mL)	369.85^a^	401.22^b^	420.56^b^	461.50^c^	4.293	<0.001
CAT (U/mL)	25.37^a^	28.77^b^	32.93^c^	35.38^c^	0.552	<0.001
MDA (nmol/mL)	5.13^c^	4.48^b^	4.14^ab^	3.63^a^	0.118	0.007

SOD, superoxide dismutase; CAT, catalase; T-AOC, total antioxidant capacity; GSH-Px, glutathione peroxidase; MDA, malondialdehyde. ^a–d^Means within same row carrying different superscript letters indicate significant differences (*p* < 0.05).

### 3.6. Effects of dietary DOL on barrier function gene expression in small intestine

**Figure 3:**
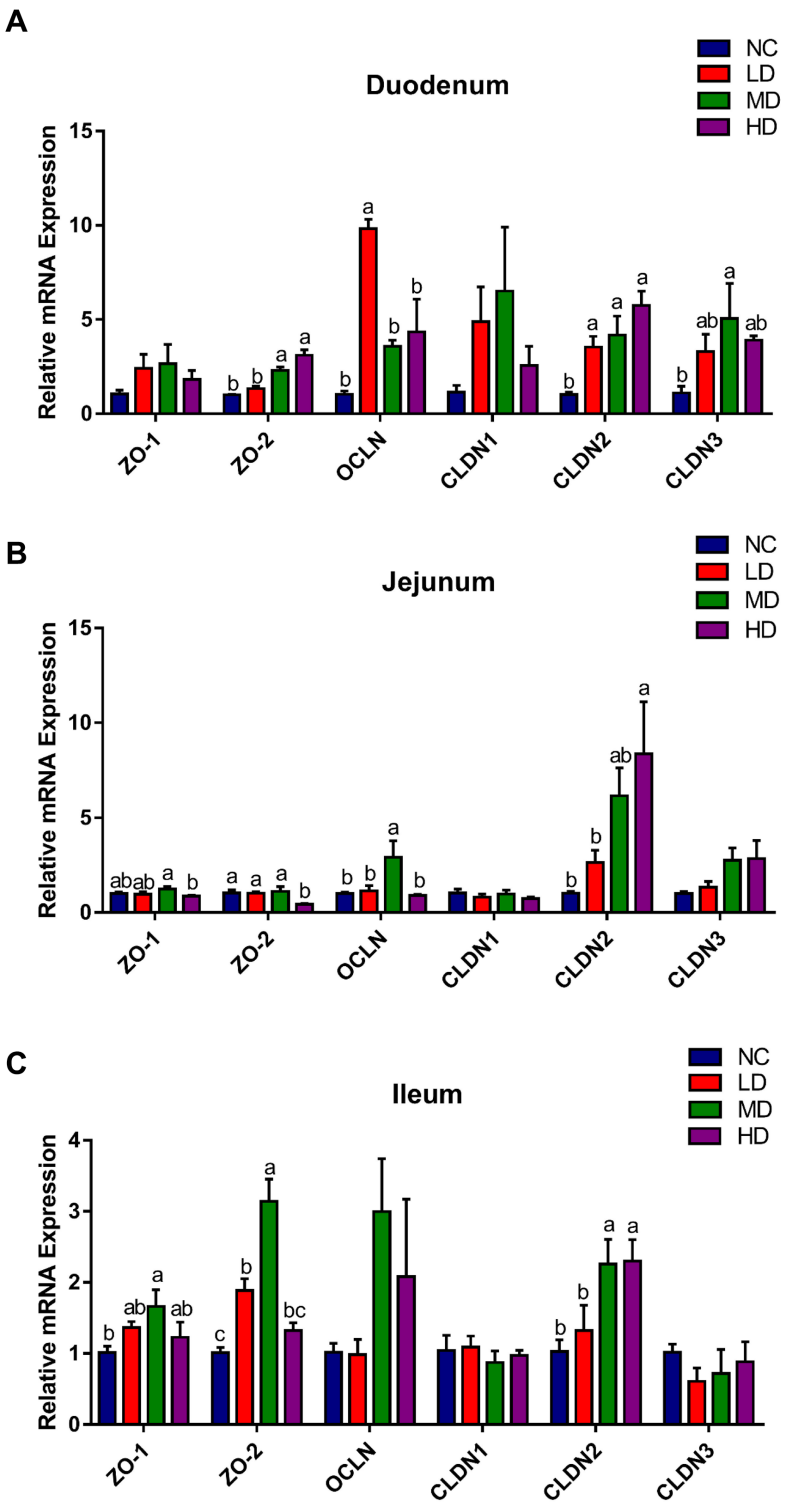
The effects of DOL supplementation on tight junction-related genes expression in the **(A)** duodenum, **(B)** jejunum, and **(C)** ileum of 70-day-old broilers. Values are represented as the mean ± SE. ^a-c^ Means among groups with different letters indicate significant differences (*p* < 0.05).

As illustrated in Figure 3, the expression of *ZO-2* and *CLDN2* in duodenum of MD and HD groups was significantly (*p* < 0.05) elevated, while LD group significantly (*p* < 0.05) increased the expression of *OCLN* and *CLDN2*, compared to the NC group. In jejunum, the expression of *OCLN* and *CLDN2* was significantly (*p* < 0.05) improved in MD and HD groups, respectively. In addition, significant (*p* < 0.05) higher expressions of *ZO-1*, *ZO-2*, and *CLDN2* were observed in ileum of MD group, and *ZO-2* and *CLDN2* were also significantly (*p* < 0.05) higher expressed in the LD and HD group, respectively, compared to those in control group.

### 3.7. Effects of dietary DOL on antioxidant-related gene expression in small intestine

**Figure 4:**
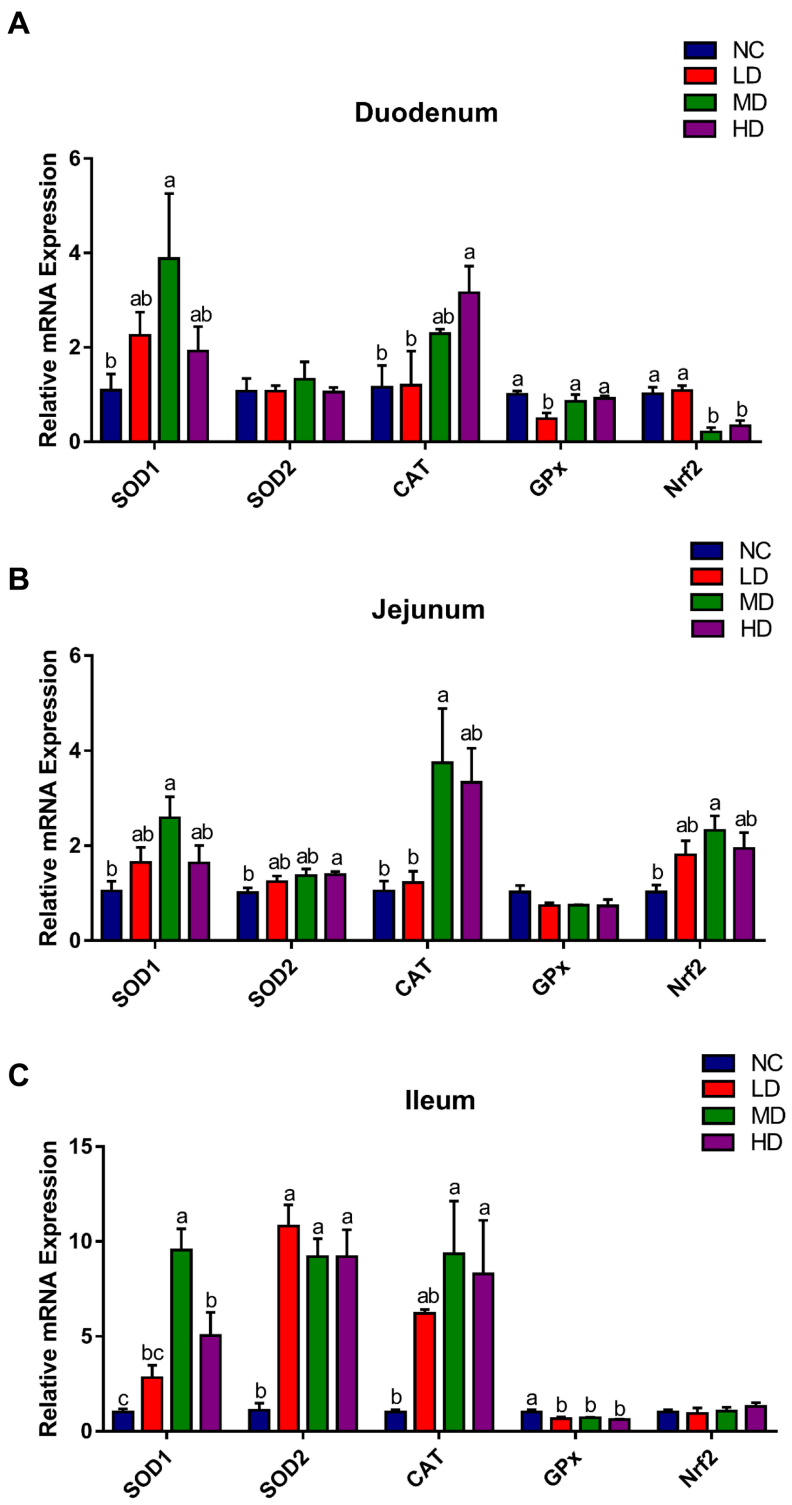
The effects of DOL supplementation on antioxidant-related gene expression in **(A)** duodenum, **(B)** jejunum, and **(C)** ileum of 70-day-old broilers. Values are represented as the mean ± SE. ^a-c^Means among groups with different letters are significantly different (*p* < 0.05).

Antioxidant-related genes expression profile in the small intestine is displayed in Figure 4. Compared with the control group, the expression of duodenal *SOD1* and *CAT* was significantly (*p* < 0.05) higher in MD and HD groups, respectively, while the expression of *Nrf2* was significantly (*p* < 0.05) decreased. 5% DOL supplementation (MD) significantly (*p* < 0.05) increased the jejunal *SOD1*, *CAT*, and *Nrf2* gene expression. Moreover, *SOD1*, *SOD2*, and *CAT* expression in ileum was significantly (*p* < 0.05) improved in MD and HD groups.

### 3.8. Effects of DOL supplementation on cecal microflora

**Figure 5:**
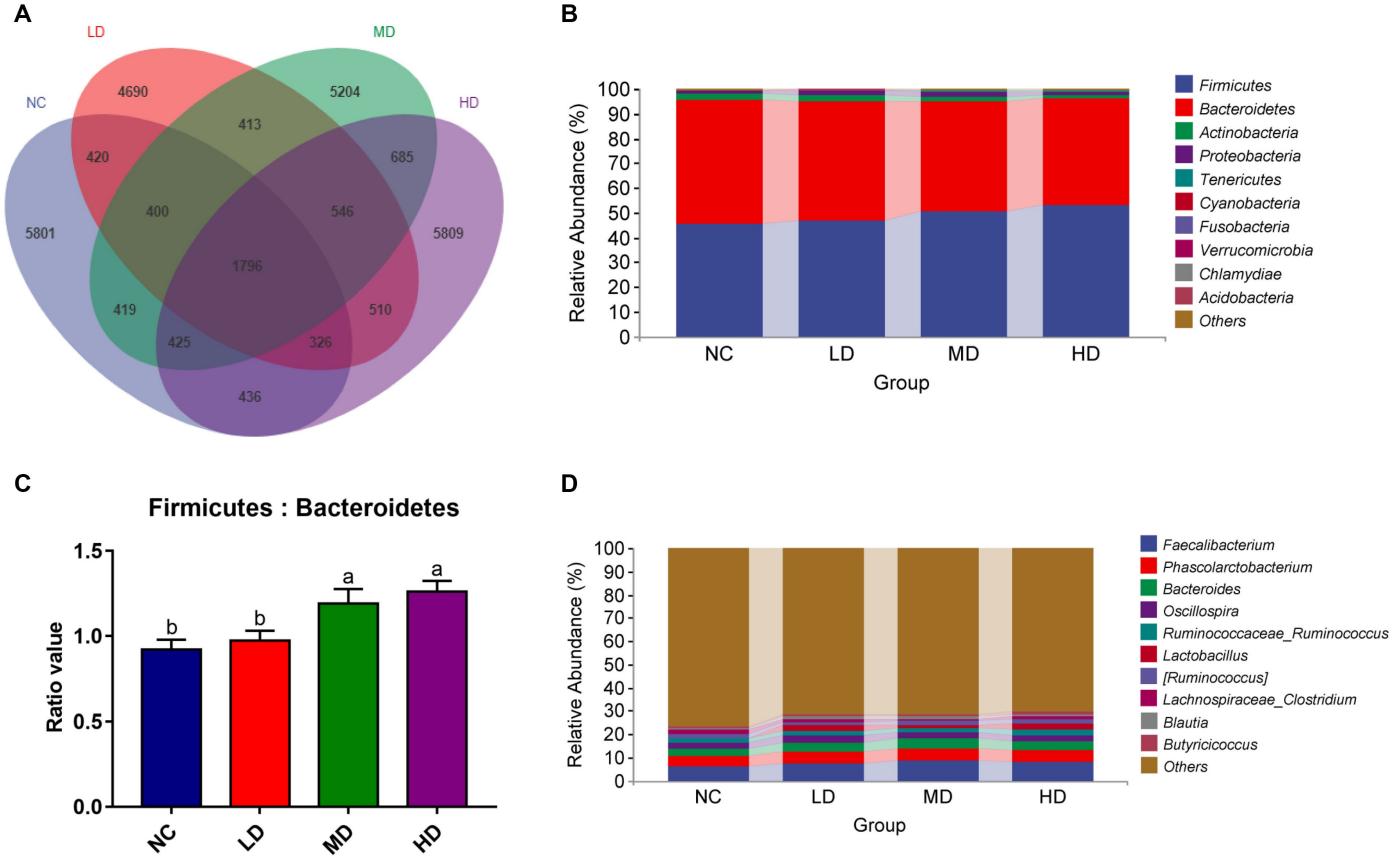
Summary of the microbial communities in the cecal contents of broilers on day 70. **(A)** Venn diagram summarizing the numbers of common and unique ASVs in the microflora, **(B)** relative abundances of the top 10 bacterial phyla in each group, **(C)** the ratio of Firmicutes to Bacteroidetes, **(D)** relative abundances of the top 10 bacterial genera in each group. ^a,b^Means among groups without the same letter are significantly different (*p* < 0.05).

We collected the cecal contents of 48 experimental broiler chickens and performed 16S rRNA sequencing to investigate the changes of microbial composition resulted from DOL supplementation. A total of 3,737,627 tags were acquired, after quality filtering, denoising, merging, and chimera removal. Each broiler’s cecal sample yielded an average of 55572.49 tags, and the estimated goods coverage for all cecal samples was greater than 99.1%. A Venn diagram showed that there were 1,796 ASVs of cecal microbiota shared among all birds; among these, the NC, LD, MD, and HD birds possessed 5,801, 4,690, 5,204, and 5,809 ASVs, respectively (Figure 5A).

At the phylum level, there were 10 phyla identified from four groups as showed in Figure 5B. With a selection of the relative top 5 (>1% of total ASVs) phyla, *Firmicutes* (36.95%–62.27%), *Bacteroidetes* (32.56%–57.26%), *Actinobacteria* (0.06%–14.66%), and *Proteobacteria* (0.25%–8.59%), and *Tenericutes* (0.10%–1.87%) dominated the cecum microbiota, representing over 99% of the total sequences. DOL dietary supplementation increased the relative abundances of *Firmicutes*, *Proteobacteria*, and *Tenericutes*, concurrent with reductions of *Bacteroidetes* and *Actinobacteria*, compared to the NC group. As a result, the ratio of *Bacteroidetes* to *Firmicutes* exhibited a significant increase in DM and DH groups, compared with the NC group (Figure 5C). The composition of cecal microflora at the genus level is presented in Figure 5D. In all groups, *Faecalibacterium* (1.36%–16.86%), *Phascolarctobacterium* (1.77%–9.31%), *Bacteroides* (1.11%–7.66%), *Oscillosspira* (0.79%–4.45%), *Ruminococcus* (0.53%–3.35%), and *Lactobacillus* (0.04%–14.62%) were the most prevalent genera, and their relative abundances were all higher in three treatment groups than in the NC group.

**Figure 6:**
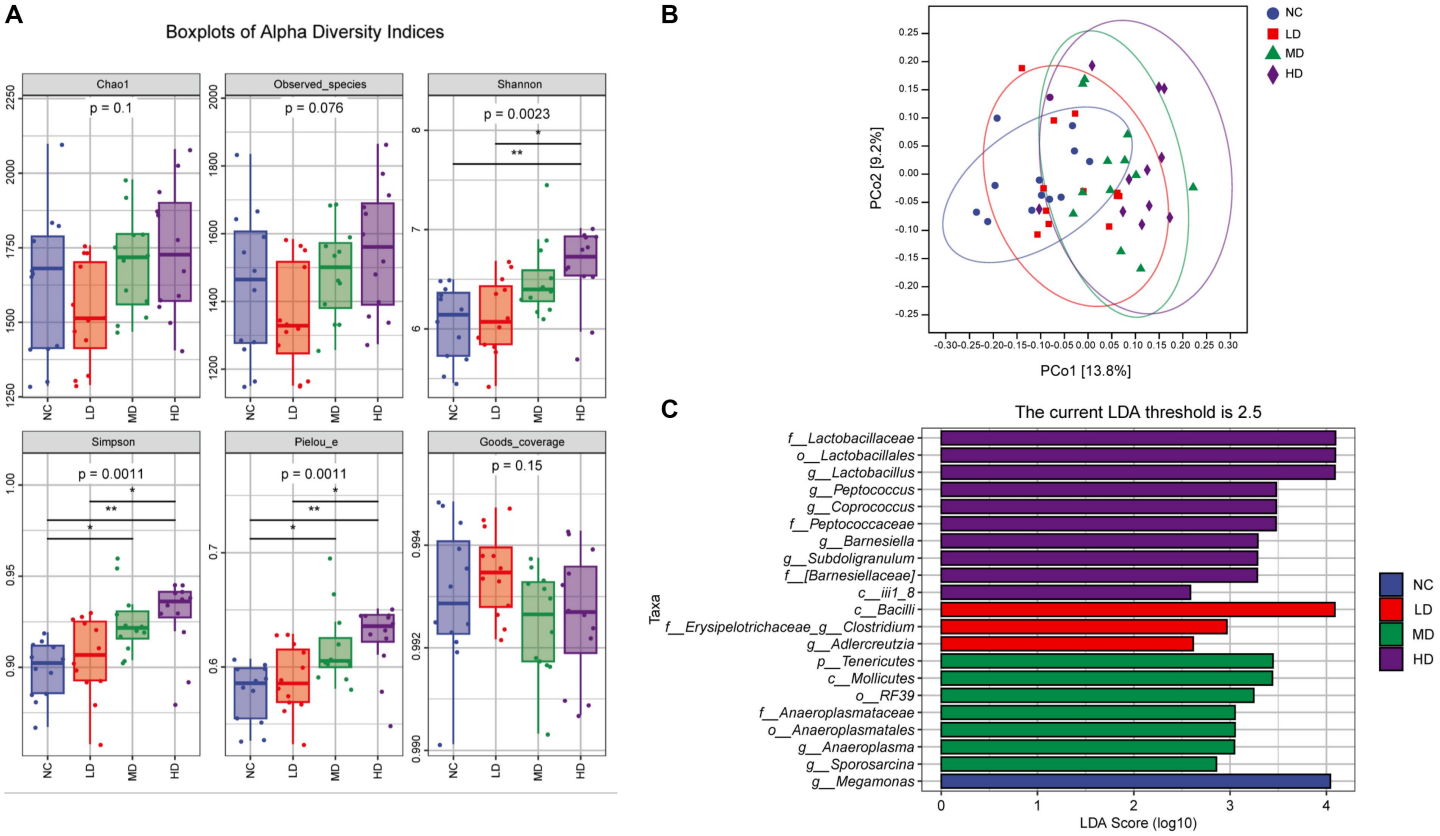
The effects of DOL on cecal microbial community diversity of the broilers on day 70. **(A)** The alpha diversity indexes, **(B)** Principal coordinate analysis (PCoA) plot using Bray Curtis distance, **(C)** Histogram shows linear discriminant analysis (LDA) scores of taxa differentially abundant between different groups. Statistical analyses were performed using linear discriminant analysis effect size (LEfSe).

Alpha diversity analysis indicated that the values of Chao 1 and observed species indexes of groups from high to low were as follows: HD, MD, NC, and LD group, but the differences among groups were not statistically significant (Dunn’s test, Figure 6 A; *p* > 0.05). Compared to the NC and LD groups, the Shannon, Simpson, and Pielou_e parameters in the HD group were all increased, and differences were statistically significant (*p* < 0.05). Additionally, the MD group also presented significantly higher Simpson and Pielou_e indexes when compared with those of the NC group (*p* < 0.05). Figure 6B illustrates the results of beta diversity analysis. The PCoA with Bray Curtis complementary algorithm showed that samples from the MD and HD groups were concentrated and relatively clustered in the first and fourth quadrants, while samples from the LD group were located in the third and fourth quadrants, and samples from NC group were in the second and third quadrants. This suggested that DOL diet changed the intestinal microbiota structure and led to a significant difference in beta-diversity (PERMANOVA test, *p* < 0.001).

Furthermore, the LEfSe analysis was conducted to further identify the most differentially abundant taxa between groups based on LDA. From phylum to genus, a total of 21 phylotypes were detected as high-dimensional biomarkers with LDA scores > 2.5 (Figure 6C). Interestingly, genera *Lactobacillus*, *Peptococcus*, *Coprococcus*, *Barnesiella*, and *Subdoligranulum* were biomarkers in HD group, whereas *Clostridium* and *Adlercreutzia* in LD group, *Tenericutes*, *Anaeroplasma* and *Sporosarcina* in MD group, and *Megamonas* in NC group were the predominant bacterial strains.

### 3.9. Cecal SCFA concentrations and correlation analysis

**Figure 7:**
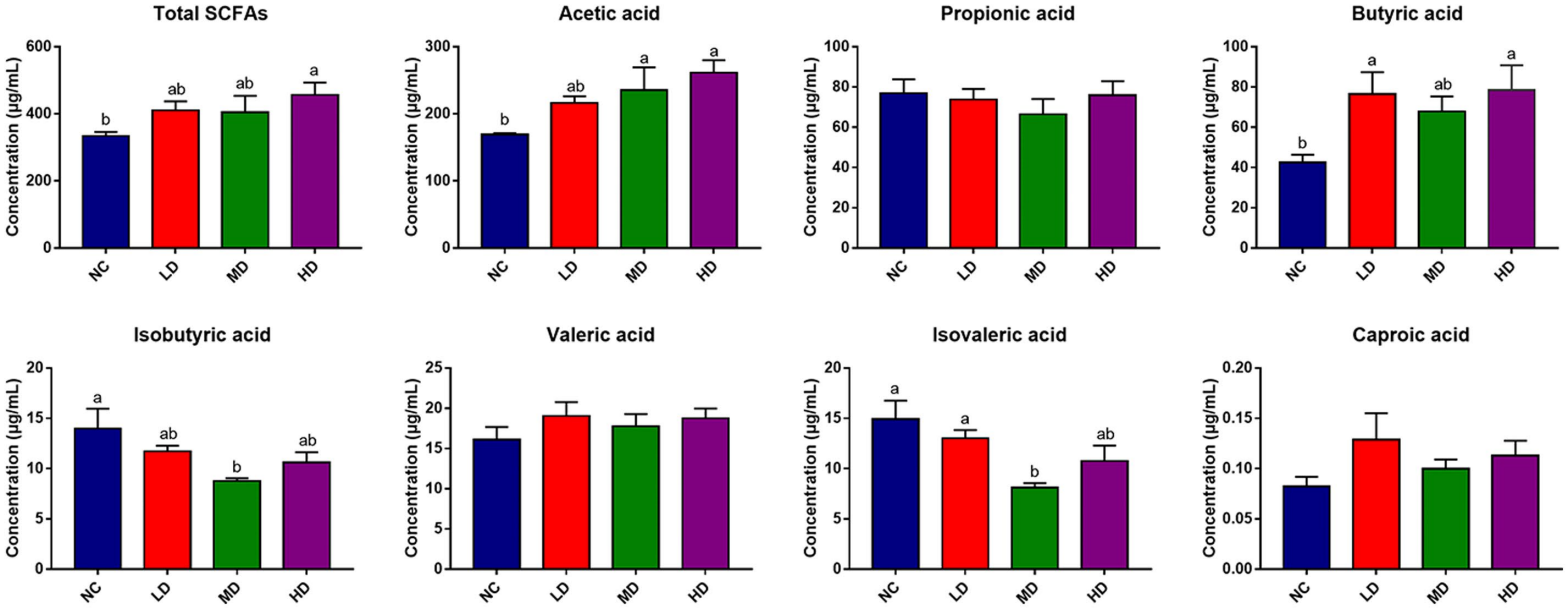
Cecum SCFAs concentration of broilers. ^a,b^Means with different superscripts over the bar indicate differ significantly (*p* < 0.05).

SCFAs concentrations in cecal contents were also determined to assess the effects of DOL addition on SCFA production, which is displayed in Figure 7. Notably, dietary DOL supplementation significantly (*p* < 0.05) increased the concentrations of total SCFAs, acetic acid, and butyric acid in the HD group compared to the control group. The concentrations of acetic acid in MD group, and butyric acid in LD group were also significantly higher (*p* < 0.05) than those in control group. In addition, the concentrations of isovaleric acid and isobutyric acid were significantly (*p* < 0.05) lower in MD group than control group. There were no significant differences in the isobutyric acid, valeric acid, and caproic acid contents found among groups.

**Figure 8:**
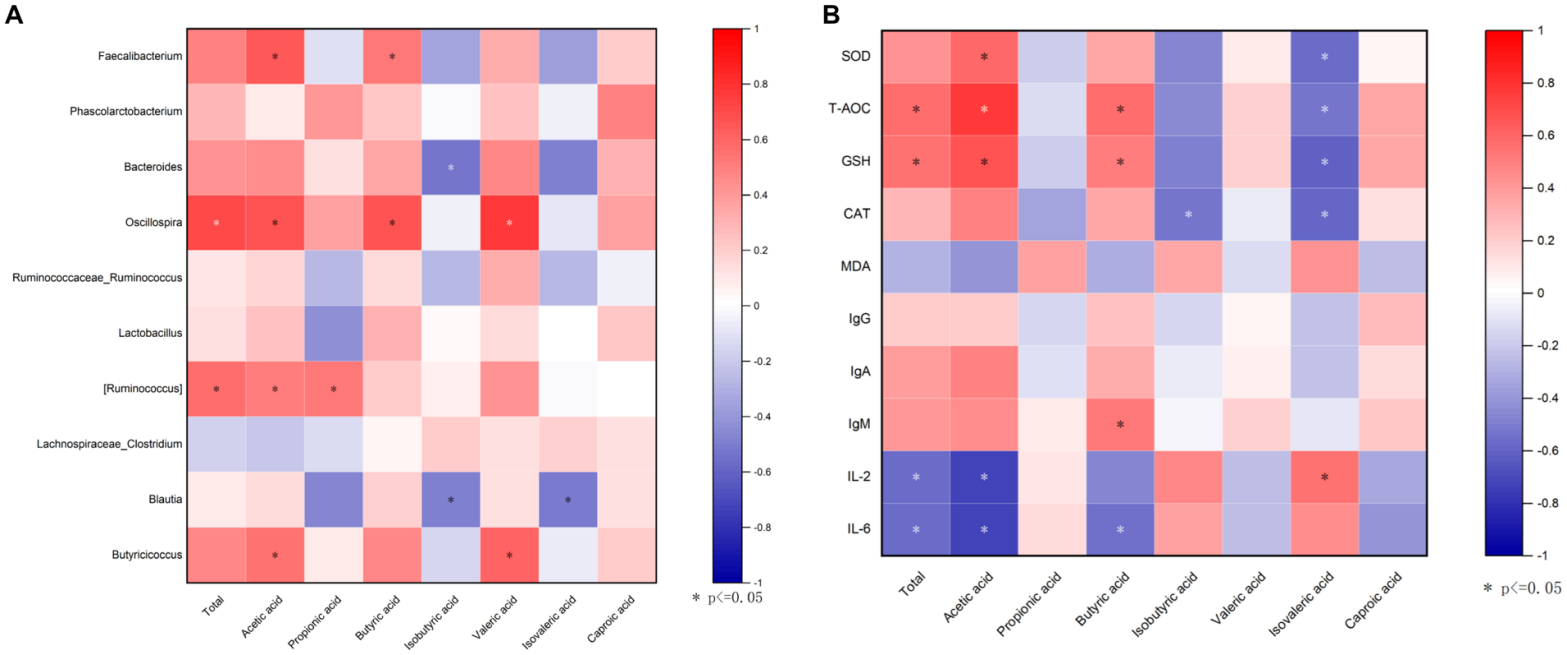
Correlation analysis. **(A)** A correlogram showing association between cecal SCFAs concentrations and the top 15 abundant cecal microbial genera; **(B)** Heatmap of Spearman’s correlation between SCFAs and antioxidant capability, and immune indexes in the serum. The spearman’s correlation coefficient is denoted by the color of the heatmap, with red indicating a positive correlation and bule indicating a negative correlation. Color depth indicates the strength of correlation; the asterisk represents a statistically significant correlation (*p* < 0.05).

To explore whether the microbiota was associated with SCFAs, a correlation analysis was carried out. Figure 8A that was generated based on the Spearman correlation coefficients, showing the relationships between the top 10 cecal genera and the cecal SCFAs changed with DOL supplementation. Among them, the relative abundances of *Faecalibacterium* and *Oscillospira* were also positively correlated with butyric acid and acetic acid (*p* < 0.05); *Oscillospira* was also positively associated with total SCFAs and valeric acid (*p* < 0.05); *Ruminococcus* was positively associated with acetic acid and propionic acid (*p* < 0.05); *Butyricicoccus* was positively associated with acetic acid and valeric acid (*p* < 0.05); whereas *Bacteroides* and *Blautia* were negatively related to isobutyric acid (*p* < 0.05). The potential relationships between cecal SCFAs concentrations and antioxidant indexes, and immune indexes were also studied. It can be seen in Figure 8B, acetic acid and butyric acid were positively correlated with T-AOC and GSH, and were inversely related correlated with IL-2 and IL-6 (*p* < 0.05); butyric acid was also positively correlated with IgM (*p* < 0.05); moreover, the total SCFAs was positively related with T-AOC and GSH, while negatively related with IL-2 and IL-6 (*p* < 0.05); isovaleric acid was negatively associated with antioxidant indexes (*p* < 0.05).

## 4. Discussion

Although *D. officinale* leaves (DOL) are not typically considered to use as a source of medicine, recently, studies have shown that DOL exerts various biological activities, especially immunomodulation and antioxidant. In the current study, we examined the effects of dietary DOL supplementation on broiler growth performance, antioxidant status, immune response, intestinal barrier function, and cecal microbiota. The observed considerable increase in final BW and ADG, as well as a decrease in F/G, compared with the NC group, provided evidence for the efficacy of 1% DOL supplementation in increasing nutrient utilization in broilers. No significant difference was found between the MD and NC group in terms of growth and feed utilization, indicating that the 5% additive dose did not influence the broiler growth performance. Since the DOL is usually discarded as a by-product in the “shihu” processing industry, the application of DOL as a feed additive can lower the cost of chicken breeding and produce high-quality broilers on the premise that they have no negative effects on animal growth. Contrarily, the reduction in daily growth observed in the HD group was probably due to the higher DOL supplementation rate (10%), which affected the nutrient levels of the diet and made the diet unable to satisfy the nutritional needs of broilers. Additionally, the deterioration of feed palatability caused a decline in feed intake, further resulting in inadequate nutrition and subpar growth performance. Little information is available on the effects of using DOL as a feed additive on animal growth performance. These data suggested that the appropriate dosage of DOL might be a potential candidate to enhance the broiler growth.

Intestinal morphology is closely associated with nutrient absorption in animals. The VH, CD, and their ratio (VH:CD) are always used as important indicators for assessing the physiological function and injury degree of intestinal tissue (Kapadia and Baker, 1976; Zhang C. et al., 2019). Generally, longer intestinal villi and lower crypt depth represent better intestinal structure and function status, contributing to greater digestive and absorptive capacity, as well as resistance to disease (Viveros et al., 2011; Munyaka et al., 2012). In our experiment, adding appropriate dosages of DOL to the diet had positive effects on intestinal morphology, as evidenced by increased VH and decreased CD in the duodenum and elevated VH in the ileum of supplemented broilers. Similar to our results, Xie et al. found that oral administration of *Dendrobium huoshanense* polysaccharide could significantly increase the VH:CD ratio in the three regions of the small intestine in mice (Xie et al., 2019). Additionally, the microporous resin extract of DOL has been demonstrated to reduce the CD, and markedly elevate the VH and the VH:CD value in the duodenum and ileum of hyperuricemia rats, indicating a potential role in maintaining the structural integrity and function of the intestinal barrier (Li et al., 2023).

The tight junctions (TJs), which can stop bacteria and toxins from entering the bloodstream, are required to maintain the intestinal mucosal barrier (Ballard et al., 1995). Additionally, the integrity of epithelial cells and barrier function of the entire intestine is determined by the permeability of the cell-connecting TJs, consisting of several vital proteins like occludin, claudins, and zonula occludens (ZOs; Fanning et al., 1998; Turner, 2006), indices of which were assessed in the present study. Claudins (translated from *CLDNs*) consists of more than 24 members of a gene family and is considered to be a critical component of the tight-junction structural skeleton, involving in barrier formation and paracellular selective permeation (Fujibe et al., 2004). Occludin (translated from *OCLN*) offers structural integrity and assembly of TJs, and the hurdle functions of the epithelial barrier depends on its sealing property (Turner, 2009). It has been reported in the literature that knockdown of *occludin* may lead to increased paracellular permeability to macromolecules, and in intestinal permeability disorders, the expression of *occludin* was also markedly reduced (Al-Sadi et al., 2011). ZO-1 and ZO-2 are critical for junction assembly (McNeil et al., 2006) and permeability (Hernandez et al., 2007), respectively, and cell could not form the TJs in their absence (Umeda et al., 2006). Earlier study had found the increased intestinal *ZO-1* and *occludin* expression in rats with macroporous resin extract of DOL administration (Deriaz and Maroudy, 1986), and *D. officinale* ultrafine powder had also been confirmed to markedly enhance the *occludin*, *claudin-1*, and *ZO-1* gene expression in rat colon (Zheng et al., 2021), suggesting that *D. officinale* could enhance the gut epithelial integrity. In line with these findings, we observed increased *ZO-1*, *ZO-2*, *OCLN* expression levels, and *CLDN* in the three segments of broiler’s small intestine, especially in the duodenum, after adding DOL. Collectively, our findings and some previous studies revealed that supplementing DOL to the diet helped to protect and maintain the structural and functional integrity of the intestinal barrier via regulating the mRNA expressions of tight junction proteins.

DOL existed helpful role on the immune function of broilers in our study. The immune organ index is a preliminary indicator reflecting the status of animal’s immune function. Here, the relative weight of immune organs, including the thymus, spleen, and bursa of Fabricius, tended to increase in broilers fed with DOL diets, and thymus index in HD was significantly increased than NC (*p* < 0.01). Immunoglobulins and cytokines play an important regulatory role in immune functions and are always used to evaluate the immune status of birds (Zhan et al., 2019). According to our results, in DOL supplementation broilers, the serum contents of IgG, IgA, and IgM were higher than those in the NC group, and the serum levels of IL-2 and IL-6 were dose-dependently reduced. These results suggested that DOL had anti-inflammatory and immunomodulatory effects on broilers. A previous *in vitro* study reported that *D. officinale* leaf polysaccharide (DOLP) reduced the damage caused by lipopolysaccharide (LPS) in human gastric epithelium GES-1 cell by preventing the release of tumor necrosis factor-α (TNF-α) and IL-6 (Yang et al., 2020). Recently, DOLP had also been proven to restore immune organ atrophy and serum IgA decrease, and to decrease the expression of TNF-α, IL-6, and IL-1β in immune deficiency mice (Xie et al., 2022). In light the aforementioned findings, it could be speculated that the modulation of immune response of broilers caused by dietary DOL possibly related to the immunomodulatory function of the polysaccharide compound in leaves.

Within cells, common metabolic activities continuously generate free radicals, which when in excess can trigger oxidative stress. This is a detrimental process that severely damages all biomolecules, thereby affecting cellular activities and even leading to cell death and disease (Amir Aslani and Ghobadi, 2016). Biological systems are equipped with highly protective antioxidant systems include both enzymatic antioxidants and non-enzymatic antioxidants, which are capable of neutralizing excessive free radicals and defending the cells from their damaging impacts (Rahman, 2007; Pham-Huy et al., 2008). The antioxidant enzymes, including superoxide (O_2_^−^) scavenger SOD and hydrogen peroxide (H_2_O_2_) scavengers GSH-Px and CAT, are considered to play a non-negligible defense role in scavenging free radicals (Zhang et al., 2014). The total antioxidant status is assessed by T-AOC, while the degree of oxidative stress is reflected by MDA, a terminal metabolite produced during free radical-induced lipid peroxidation (Cao et al., 2016; Liu et al., 2021). As displayed in the current study, slight or significant increments of SOD, T-AOC, and CAT levels, along with a reduction of MDA content, were observed in the serum of DOL-fed broilers. Moreover, the DOL also upregulated the mRNA expressions of antioxidant-related genes (*SOD1*, *SOD2*, and *CAT*) in the small intestine tissues of broilers. Natural flavonols, flavones, flavanones, and other classes of compounds have been proven to exhibit strong antioxidant capacities (Goncalves et al., 2017). Sufficient literature had proved that the extract of DOL contained a high content of flavonoids and had potent antioxidant activities (Zhang et al., 2017). Additionally, polysaccharides from DOL have also been confirmed to attenuate ethanol-induced gastric mucosal injury by drastically lowering ROS generation and increasing SOD and T-AOC levels (Ke et al., 2020). Therefore, the antioxidant function of the flavonoids and polysaccharides present in DOL might be responsible for the enhanced antioxidant status of DOL supplemented broilers.

The gut microbiota has a range of advantageous impacts on their hosts, including nutrition digestion and defense against pathogen invasion, and contributes to maintain the normal physiological processes of the intestine (Chang et al., 2020; Slizewska et al., 2020). Numerous studies on the gut microbiota of normal broilers have reported that *Firmicutes* and *Bacteroidetes* were the predominant intestinal phyla (Chen et al., 2020; Das et al., 2020), which was also observed in the cecal contents of all broilers in our study. *Firmicutes* and *Bacteroidetes* have been confirmed to participate in the degradation and metabolism of nutrients, such as carbohydrate and indigestible complex polysaccharides, promoting nutrient absorption and energy capture from feed ingredients in animals (Liu et al., 2018; Ouyang et al., 2019). In the current study, these two phyla occupied greater than 95% of the microbiota in cecum, which is the major microbial fermentative organ in poultry (Vasai et al., 2014), suggesting their importance in feed utilization and host metabolism. Additionally, broilers fed with DOL had an increased abundance of *Firmicutes* and a decreased abundance of *Bacteroidetes* in the comparison with the control group, as well as a greater *Firmicutes* to *Bacteroidetes* ratio that was correlated with a bacterial profile with a higher capacity for energy harvesting (Singh et al., 2012; Bervoets et al., 2013). At genus level, *Faecalibacterium* and *Phascolarctobacterium* were the main categories in cecum and showed higher enrichment in DOL-supplemented broilers. *Faecalibacterium* is one of the main producers of butyric acid and is considered a very important bacterial indicator of a health gut (Li et al., 2019; Ye et al., 2019). The butyrate-producing bacterium of species *Faecalibacterium_prausnitzii*, which is associated with good feed efficiency (Kameyama and Itoh, 2014), accounted for the largest proportion of bacteria species and showed the highest relative abundance in MD group in this study. It has been demonstrated that *Phascolarctobacterium* are capable of synthesizing propionate from sugars. The other predominant genus detected in cecum was *Bacteroides*, which helps chickens enhance weight gain and growth performance (Chang et al., 2016). Thus, we could conclude that the highest abundance of *Bacteroides* observed in LD group might be related to the highest ADG of LD broilers. Notably, LEfSe’s analysis results further detected the predominance of *Lactobacillus* in HD group. *Lactobacillus* is a beneficial type of bacteria that has a potent ability to adhere to epithelial tissues and prevent the colonization of undesirable bacteria, which can improve intestinal health and augment the intestinal barrier effect (Gao et al., 2017; Xue et al., 2017). It can also ferment carbohydrates to produce lactic acid, which contributes to maintaining health and regulating immune functions. *Anaeroplasma*, a probiotic with anti-inflammatory potential, may stimulate IgA-secreting plasma cells in the small intestine and increase the level of mucosal IgA (Beller et al., 2020), was identified as a biomarker in MD group. On the other hand, in this study, the Shannon, Simpson, and Pielou_e indexes of the cecal microflora in the MD and HD groups were significantly higher than control group, but the Chao1 and observed species indexes were not significantly different, suggesting that the addition of medium- and high-dose DOL could significantly improve the diversity and evenness of broiler cecal microflora but had little effect on richness. In accordance with our results, Zhang et al. also described that the addition of DOP could obviously improve the diversity of gut microbiota and elevate the abundance of bacteria, including *Bacteroides* and *Lactobacillus*, in intestine of colitis mice (Zhang et al., 2020). Similarly, *D. officinale* was discovered to increase the diversity of intestinal mucosal flora, and encourage the abundance of *Ochrobactrum* in mice fed a high-fat diet (Li et al., 2021). Higher abundance and diversity have been reported to favor the maintenances of the dynamic balance of microecosystem, intestinal health, and normal physiological functions (Diaz Carrasco et al., 2018; Dhillon et al., 2019). Recent studies also reported that oral administration of DOL polysaccharides could raise the relative abundances of total bacteria, especially probiotics (e.g., *Bacteroides*, *Lactobacillus*, and *Lachnospiraceae*) to improve the gut microbiota in mice (Fang et al., 2022; Xie et al., 2022). Overall, these results revealed that dietary DOL supplementation might alter the relative abundances and structure of cecum microbiota in broilers and, mostly, to promote the colonization of beneficial bacteria.

As is well known, one of the richest metabolites produced by microbial fermentation, SCFAs are well established to be crucial for the maintenance of intestinal barrier function, immune function and metabolism (Tan et al., 2014). In the current study, we found that the broilers supplemented with DOL had higher contents of SCFAs, particularly acetic acid and butyric acid in the cecal contents than broilers fed a control diet, suggesting the effects of DOL on modulating the fermentation characteristics of cecal microbiota. SCFAs are found to be produced mainly in *Firmicutes* (Videnska et al., 2014; Chakraborti, 2015). According to the results of Spearman’s rank correlation analysis, some butyrate-producing flora belonging to *Firmicutes*, i.e., *Faecalibacterium* (Ye et al., 2019) and *Oscillospira* (Konikoff and Gophna, 2016; Gophna et al., 2017) that had greater enrichment in DOL groups were positively correlated with cecal acetic acid or butyric acid. Previously published literature has stated that DOLP could improve the SCFAs levels, which might be due to the change in intestinal flora microstructure in mice (Fang et al., 2022; Xie et al., 2022). Taken together, these results further confirmed that the increase of SCFAs concentrations induced by dietary DOL supplementation was the result of altered microbiota composition, e.g., enhanced intestinal colonization of SCFA-producing bacteria.

The importance of SCFAs, especially butyric acid, for gut health is being recognized more and more, but they may also enter the systemic circulation and have an impact on peripheral tissue functions or metabolism (Van den Abbeele et al., 2011). The positive correlations of acetic acid and butyric acid with the antioxidant enzymes (T-AOC and GSH-Px) activities and IgM level and negative associations with cytokines IL-2 and IL-6 displayed in our spearman correlation analysis imply that this SCFA would be an important contributor to host antioxidant capability. This is in line with the study of Miao et al. (2022), who also described the positive relationship between SCFAs and intestinal antioxidant indexes. Butyric acid is the preferred energy source for enterocytes and modulates a variety of physiological processes, including oxidation (Fu et al., 2019), inflammation (Onrust et al., 2015), and barrier integrity (Salvi and Cowles, 2021). Acetic acid is modulator of immunity and redox signaling. Therefore, the increased production of SCFAs may contribute to the observed changes in antioxidant and immune indexes, which enhance host antioxidant capability and immune function.

## 5. Conclusion

In summary, the results of the present study indicated that *D. officinale* leaves supplementation in broiler diets improved the activities of antioxidant enzymes (SOD, CAT, and GSH-Px) and IgA level in serum and showed positive effects on gut health via enhancing small intestinal antioxidant-related gene expression, improving intestinal morphology, increasing SCFAs production, and regulating the cecum microbial communities. Under the experimental conditions, 1% and 5% DOL supplementation to the feed would be more appropriate because they could maintain the broilers’ growth performance and reduce feed costs. Our results will provide a scientific basis for DOL’s application as a feed additive in broiler feed in the future.

## Data availability statement

The datasets presented in this study can be found in online repositories. The names of the repository/repositories and accession number(s) can be found at: https://www.ncbi.nlm.nih.gov/, PRJNA942368.

## Ethics statement

Animals used in this study were raised and slaughtered in accordance with the National Standard of Laboratory Animal Guideline for ethical review of animal welfare (GB/T 35892-2018), issued by General Administration of Quality Supervision, Inspection and Quarantine of the people’s Republic of China and Standardization Administration of the People’s Republic of China. All experiment procedures were approved by the Animal Care and Use Committee of Zhejiang Academy of Agricultural Sciences (Hangzhou, China). The study was conducted in accordance with the local legislation and institutional requirements.

## Author contributions

WZ: Data curation, Writing – original draft. YC: Data curation, Writing – review & editing. YT: Funding acquisition, Methodology, Writing – review & editing. YW: Formal analysis, Software, Writing – review & editing. JD: Formal analysis, Software, Writing – review & editing. XY: Funding acquisition, Resources, Writing – review & editing. LL: Conceptualization, Methodology, Project administration, Writing – review & editing. CS: Project administration, Visualization, Writing – review & editing.

## Funding

The author(s) declare financial support was received for the research, authorship, and/or publication of this article. This work was supported by the Key Research and Development Program of Zhejiang Province (no. 2021C02034) and Zhejiang Provincial Special Commissioner Team Projects of Science and Technology (no. Xianju Chicken Industry, 2020–2024).

## Conflict of interest

The authors declare that the research was conducted in the absence of any commercial or financial relationships that could be construed as a potential conflict of interest.

## Publisher’s note

All claims expressed in this article are solely those of the authors and do not necessarily represent those of their affiliated organizations, or those of the publisher, the editors and the reviewers. Any product that may be evaluated in this article, or claim that may be made by its manufacturer, is not guaranteed or endorsed by the publisher.

## Footnotes

^1^ www.PremierBiosoft.com

^2^ https://docs.qiime2.org/2019.4/tutorials/

## References

[ref1] Al-Sadi R. Khatib K. Guo S. Ye D. Youssef M. Ma T. (2011). Occludin regulates macromolecule flux across the intestinal epithelial tight junction barrier. Am. J. Physiol. Gastrointest. Liver Physiol. 300, G1054–G1064. doi: 10.1152/ajpgi.00055.2011 21415414 PMC3119114

[ref2] Aminov R. I. (2010). A brief history of the antibiotic era: lessons learned and challenges for the future. Front. Microbiol. 1:134. doi: 10.3389/fmicb.2010.00134 21687759 PMC3109405

[ref3] Amir Aslani B. Ghobadi S. (2016). Studies on oxidants and antioxidants with a brief glance at their relevance to the immune system. Life Sci. 146, 163–173. doi: 10.1016/j.lfs.2016.01.014 26792059

[ref4] Aoi W. Iwasa M. Marunaka Y. (2021). Metabolic functions of flavonoids: from human epidemiology to molecular mechanism. Neuropeptides 88:102163. doi: 10.1016/j.npep.2021.102163 34098453

[ref5] Bai R. Yao C. Zhong Z. Ge J. Bai Z. Ye X.. (2021). Discovery of natural anti-inflammatory alkaloids: potential leads for the drug discovery for the treatment of inflammation. Eur. J. Med. Chem. 213:113165. doi: 10.1016/j.ejmech.2021.113165 33454546

[ref6] Ballard S. T. Hunter J. H. Taylor A. E. (1995). Regulation of tight-junction permeability during nutrient absorption across the intestinal epithelium. Annu. Rev. Nutr. 15, 35–55. doi: 10.1146/annurev.nu.15.070195.000343 8527224

[ref7] Beller A. Kruglov A. Durek P. von Goetze V. Werner K. Heinz G. A.. (2020). Specific microbiota enhances intestinal IgA levels by inducing TGF-beta in T follicular helper cells of Peyer's patches in mice. Eur. J. Immunol. 50, 783–794. doi: 10.1002/eji.201948474 32065660

[ref8] Bervoets L. Van Hoorenbeeck K. Kortleven I. Van Noten C. Hens N. Vael C.. (2013). Differences in gut microbiota composition between obese and lean children: a cross-sectional study. Gut Pathog 5:10. doi: 10.1186/1757-4749-5-10 23631345 PMC3658928

[ref9] Bokulich N. A. Kaehler B. D. Rideout J. R. Dillon M. Bolyen E. Knight R.. (2018). Optimizing taxonomic classification of marker-gene amplicon sequences with QIIME 2's q2-feature-classifier plugin. Microbiome 6:90. doi: 10.1186/s40168-018-0470-z 29773078 PMC5956843

[ref10] Bolyen E. Rideout J. R. Dillon M. R. Bokulich N. A. Abnet C. C. Al-Ghalith G. A.. (2019). Reproducible, interactive, scalable and extensible microbiome data science using QIIME 2. Nat. Biotechnol. 37, 852–857. doi: 10.1038/s41587-019-0209-9 31341288 PMC7015180

[ref11] Callahan B. J. McMurdie P. J. Rosen M. J. Han A. W. Johnson A. J. Holmes S. P. (2016). DADA2: high-resolution sample inference from Illumina amplicon data. Nat. Methods 13, 581–583. doi: 10.1038/nmeth.3869 27214047 PMC4927377

[ref12] Cao W. Xiao L. Liu G. Fang T. Wu X. Jia G.. (2016). Dietary arginine and N-carbamylglutamate supplementation enhances the antioxidant statuses of the liver and plasma against oxidative stress in rats. Food Funct. 7, 2303–2311. doi: 10.1039/c5fo01194a 27109002

[ref13] Chakraborti C. K. (2015). New-found link between microbiota and obesity. World J Gastrointest Pathophysiol 6, 110–119. doi: 10.4291/wjgp.v6.i4.110 26600968 PMC4644874

[ref14] Chang C. L. Chung C. Y. Kuo C. H. Kuo T. F. Yang C. W. Yang W. C. (2016). Beneficial effect of *Bidens pilosa* on body weight gain, food conversion ratio, gut Bacteria and coccidiosis in chickens. PloS One 11:e0146141. doi: 10.1371/journal.pone.0146141 26765226 PMC4713076

[ref15] Chang J. Wang T. Wang P. Yin Q. Liu C. Zhu Q.. (2020). Compound probiotics alleviating aflatoxin B(1) and zearalenone toxic effects on broiler production performance and gut microbiota. Ecotoxicol. Environ. Saf. 194:110420. doi: 10.1016/j.ecoenv.2020.110420 32151861

[ref16] Chen Y. Wang J. Yu L. Xu T. Zhu N. (2020). Microbiota and metabolome responses in the cecum and serum of broiler chickens fed with plant essential oils or virginiamycin. Sci. Rep. 10:5382. doi: 10.1038/s41598-020-60135-x 32214106 PMC7096418

[ref17] Das Q. Islam M. R. Lepp D. Tang J. Yin X. Mats L.. (2020). Gut microbiota, blood metabolites, and spleen immunity in broiler chickens fed berry pomaces and phenolic-enriched extractives. Front Vet Sci 7:150. doi: 10.3389/fvets.2020.00150 33134328 PMC7188780

[ref18] Deriaz H. Maroudy D. (1986). Computerization of patient movement within a nursing unit. Soins 489-490, 13–16. 3644440

[ref19] Dhillon J. Li Z. Ortiz R. M. (2019). Almond snacking for 8 wk increases alpha-diversity of the gastrointestinal microbiome and decreases *Bacteroides fragilis* abundance compared with an Isocaloric snack in college freshmen. Curr Dev Nutr 3:nzz079. doi: 10.1093/cdn/nzz079 PMC6736066 31528836

[ref20] Diaz Carrasco J. M. Redondo E. A. Pin Viso N. D. Redondo L. M. Farber M. D. Fernandez Miyakawa M. E. (2018). Tannins and bacitracin differentially modulate gut microbiota of broiler chickens. Biomed. Res. Int. 2018:1879168. doi: 10.1155/2018/1879168 29682522 PMC5841071

[ref21] Fang J. Lin Y. Xie H. Farag M. A. Feng S. Li J.. (2022). Dendrobium officinale leaf polysaccharides ameliorated hyperglycemia and promoted gut bacterial associated SCFAs to alleviate type 2 diabetes in adult mice. Food Chem X 13:100207. doi: 10.1016/j.fochx.2022.100207 35498995 PMC9039915

[ref22] Fanning A. S. Jameson B. J. Jesaitis L. A. Anderson J. M. (1998). The tight junction protein ZO-1 establishes a link between the transmembrane protein occludin and the actin cytoskeleton. J. Biol. Chem. 273, 29745–29753. doi: 10.1074/jbc.273.45.29745 9792688

[ref24] Fujibe M. Chiba H. Kojima T. Soma T. Wada T. Yamashita T.. (2004). Thr203 of claudin-1, a putative phosphorylation site for MAP kinase, is required to promote the barrier function of tight junctions. Exp. Cell Res. 295, 36–47. doi: 10.1016/j.yexcr.2003.12.014 15051488

[ref23] Fu X. Liu Z. Zhu C. Mou H. Kong Q. (2019). Nondigestible carbohydrates, butyrate, and butyrate-producing bacteria. Crit. Rev. Food Sci. Nutr. 59, S130–S152. doi: 10.1080/10408398.2018.1542587 30580556

[ref25] Gao P. Ma C. Sun Z. Wang L. Huang S. Su X.. (2017). Feed-additive probiotics accelerate yet antibiotics delay intestinal microbiota maturation in broiler chicken. Microbiome 5:91. doi: 10.1186/s40168-017-0315-1 28768551 PMC5541433

[ref26] Goncalves S. Moreira E. Grosso C. Andrade P. B. Valentao P. Romano A. (2017). Phenolic profile, antioxidant activity and enzyme inhibitory activities of extracts from aromatic plants used in Mediterranean diet. J. Food Sci. Technol. 54, 219–227. doi: 10.1007/s13197-016-2453-z 28242919 PMC5305718

[ref27] Gophna U. Konikoff T. Nielsen H. B. (2017). Oscillospira and related bacteria—from metagenomic species to metabolic features. Environ. Microbiol. 19, 835–841. doi: 10.1111/1462-2920.13658 28028921

[ref28] Hernandez S. Chavez Munguia B. Gonzalez-Mariscal L. (2007). ZO-2 silencing in epithelial cells perturbs the gate and fence function of tight junctions and leads to an atypical monolayer architecture. Exp. Cell Res. 313, 1533–1547. doi: 10.1016/j.yexcr.2007.01.026 17374535

[ref29] International, A. (2007). Official methods of analysis of AOAC international, 18th Edn. AOAC International, Gaithersburg, MD.

[ref30] Kameyama K. Itoh K. (2014). Intestinal colonization by a Lachnospiraceae bacterium contributes to the development of diabetes in obese mice. Microbes Environ. 29, 427–430. doi: 10.1264/jsme2.ME14054 25283478 PMC4262368

[ref31] Kapadia S. Baker S. J. (1976). The effects of alterations in villus shape on the intestinal mucosal surface of the albino rat; the relationship between mucosal surface area and the crypts. Digestion 14, 256–268. doi: 10.1159/000197939 955332

[ref32] Katoh K. Misawa K. Kuma K. Miyata T. (2002). MAFFT: a novel method for rapid multiple sequence alignment based on fast Fourier transform. Nucleic Acids Res. 30, 3059–3066. doi: 10.1093/nar/gkf436 12136088 PMC135756

[ref33] Ke Y. Zhan L. Lu T. Zhou C. Chen X. Dong Y.. (2020). Polysaccharides of Dendrobium officinale Kimura & Migo Leaves Protect against Ethanol-Induced Gastric Mucosal Injury via the AMPK/mTOR signaling pathway in vitro and vivo. Front. Pharmacol. 11:526349. doi: 10.3389/fphar.2020.526349 33262700 PMC7686799

[ref34] Koljalg U. Nilsson R. H. Abarenkov K. Tedersoo L. Taylor A. F. Bahram M.. (2013). Towards a unified paradigm for sequence-based identification of fungi. Mol. Ecol. 22, 5271–5277. doi: 10.1111/mec.12481 24112409

[ref35] Konikoff T. Gophna U. (2016). Oscillospira: a central, enigmatic component of the human gut microbiota. Trends Microbiol. 24, 523–524. doi: 10.1016/j.tim.2016.02.015 26996766

[ref40] Liang J. Li H. Chen J. He L. Du X. Zhou L.. (2019). Dendrobium officinale polysaccharides alleviate colon tumorigenesis via restoring intestinal barrier function and enhancing anti-tumor immune response. Pharmacol. Res. 148:104417. doi: 10.1016/j.phrs.2019.104417 31473343

[ref36] Li H. Li H. Xie P. Li Z. Yin Y. Blachier F.. (2019). Dietary supplementation with fermented Mao-tai lees beneficially affects gut microbiota structure and function in pigs. AMB Exp 9:26. doi: 10.1186/s13568-019-0747-z PMC6379501 30778768

[ref41] Lillehoj H. Liu Y. Calsamiglia S. Fernandez-Miyakawa M. E. Chi F. Cravens R. L.. (2018). Phytochemicals as antibiotic alternatives to promote growth and enhance host health. Vet. Res. 49:76. doi: 10.1186/s13567-018-0562-6 30060764 PMC6066919

[ref38] Li L. Z. Wang X. M. Feng X. J. Liu K. Li B. Zhu L. J.. (2023). Effects of a macroporous resin extract of Dendrobium officinale leaves in rats with hyperuricemia induced by anthropomorphic unhealthy lifestyle. Evid. Based Complement. Alternat. Med. 2023:9990843. doi: 10.1155/2023/9990843 36644440 PMC9839412

[ref39] Li S. Wu D. Cao M. Yu Z. Wu M. Liu Y.. (2020). Effects of choline supplementation on liver biology, gut microbiota, and inflammation in *Helicobacter pylori*-infected mice. Life Sci. 259:118200. doi: 10.1016/j.lfs.2020.118200 32758621

[ref42] Liu B. Shang Z. Z. Li Q. M. Zha X. Q. Wu D. L. Yu N. J.. (2020). Structural features and anti-gastric cancer activity of polysaccharides from stem, root, leaf and flower of cultivated Dendrobium huoshanense. Int. J. Biol. Macromol. 143, 651–664. doi: 10.1016/j.ijbiomac.2019.12.041 31821827

[ref43] Liu S. J. Wang J. He T. F. Liu H. S. Piao X. S. (2021). Effects of natural capsicum extract on growth performance, nutrient utilization, antioxidant status, immune function, and meat quality in broilers. Poult. Sci. 100:101301. doi: 10.1016/j.psj.2021.101301 34273651 PMC8313837

[ref44] Liu Z. Wang X. Ou S. Arowolo M. A. Hou D. X. He J. (2018). Effects of *Achyranthes bidentata* polysaccharides on intestinal morphology, immune response, and gut microbiome in yellow broiler chickens challenged with *Escherichia coli* K88. Polymers 10:1233. doi: 10.3390/polym10111233 30961158 PMC6401798

[ref45] Livak K. J. Schmittgen T. D. (2001). Analysis of relative gene expression data using real-time quantitative PCR and the 2(-Delta Delta C(T)) method. Methods 25, 402–408. doi: 10.1006/meth.2001.1262 11846609

[ref37] Li X. Peng X. Guo K. Tan Z. (2021). Bacterial diversity in intestinal mucosa of mice fed with Dendrobium officinale and high-fat diet. 3 Biotech 11:22. doi: 10.1007/s13205-020-02558-x PMC7779387 33442520

[ref46] Long L. N. Zhang H. H. Wang F. Yin Y. X. Yang L. Y. Chen J. S. (2021). Effects of polysaccharide-enriched *Acanthopanax senticosus* extract on growth performance, immune function, antioxidation, and ileal microbial populations in broiler chickens. Poult. Sci. 100:101028. doi: 10.1016/j.psj.2021.101028 33647719 PMC7921867

[ref47] Lozupone C. A. Hamady M. Kelley S. T. Knight R. (2007). Quantitative and qualitative beta diversity measures lead to different insights into factors that structure microbial communities. Appl. Environ. Microbiol. 73, 1576–1585. doi: 10.1128/AEM.01996-06 17220268 PMC1828774

[ref48] Lozupone C. Knight R. (2005). UniFrac: a new phylogenetic method for comparing microbial communities. Appl. Environ. Microbiol. 71, 8228–8235. doi: 10.1128/AEM.71.12.8228-8235.2005 16332807 PMC1317376

[ref49] Martin M. (2011). CUTADAPT removes adapter sequences from high-throughput sequencing reads. EMBnet J 17:10. doi: 10.14806/ej.17.1.200

[ref50] Mavrommatis A. Giamouri E. Myrtsi E. D. Evergetis E. Filippi K. Papapostolou H.. (2021). Antioxidant status of broiler chickens fed diets supplemented with Vinification by-products: a valorization approach. Antioxidants 10:1250. doi: 10.3390/antiox10081250 34439498 PMC8389203

[ref51] McArdle B. H. Anderson M. J. (2001). Fitting multivariate models to community data: a comment on distance-based redundancy analysis. Ecology 82, 290–297. doi: 10.1890/00129658(2001)082[0290,fmmtcd]2.0.co;2

[ref52] McNeil E. Capaldo C. T. Macara I. G. (2006). Zonula occludens-1 function in the assembly of tight junctions in Madin-Darby canine kidney epithelial cells. Mol. Biol. Cell 17, 1922–1932. doi: 10.1091/mbc.e05-07-0650 16436508 PMC1415307

[ref53] Meng Y. Yu D. Xue J. Lu J. Feng S. Shen C.. (2016). A transcriptome-wide, organ-specific regulatory map of Dendrobium officinale, an important traditional Chinese orchid herb. Sci. Rep. 6:18864. doi: 10.1038/srep18864 26732614 PMC4702150

[ref54] Miao S. Hong Z. Jian H. Xu Q. Liu Y. Wang X.. (2022). Alterations in intestinal antioxidant and immune function and Cecal microbiota of laying Hens fed on coated sodium butyrate supplemented diets. Animals 12:545. doi: 10.3390/ani12050545 35268114 PMC8908843

[ref55] Munyaka P. M. Echeverry H. Yitbarek A. Camelo-Jaimes G. Sharif S. Guenter W.. (2012). Local and systemic innate immunity in broiler chickens supplemented with yeast-derived carbohydrates. Poult. Sci. 91, 2164–2172. doi: 10.3382/ps.2012-02306 22912450

[ref9001] Ng T. B. Liu J. Wong J. H. Ye X. Wing Sze S. C. Tong Y. (2012). Review of research on Dendrobium, a prized folk medicine. Appl. Microbiol. Biotechnol. 93, 1795–1803. doi: 10.1007/s00253-011-3829-7 22322870

[ref56] Onrust L. Ducatelle R. Van Driessche K. De Maesschalck C. Vermeulen K. Haesebrouck F.. (2015). Steering endogenous butyrate production in the intestinal tract of broilers as a tool to improve gut health. Front Vet Sci 2:75. doi: 10.3389/fvets.2015.00075 26734618 PMC4682374

[ref57] Ouyang E. Lu Y. Ouyang J. Wang L. Wang X. (2019). Performance and dynamic characteristics of microbial communities in multi-stage anaerobic reactors treating gibberellin wastewater. J. Biosci. Bioeng. 127, 318–325. doi: 10.1016/j.jbiosc.2018.05.017 30514617

[ref58] Peng M. J. Huang T. Yang Q. L. Peng S. Jin Y. X. Wang X. S. (2022). Dietary supplementation *Eucommia ulmoides* extract at high content served as a feed additive in the hens industry. Poult. Sci. 101:101650. doi: 10.1016/j.psj.2021.101650 35121531 PMC8814652

[ref59] Pham-Huy L. A. He H. Pham-Huy C. (2008). Free radicals, antioxidants in disease and health. Int. J. Biomed. Sci. 4, 89–96. 23675073 PMC3614697

[ref60] Rahman K. (2007). Studies on free radicals, antioxidants, and co-factors. Clin. Interv. Aging 2, 219–236. 18044138 PMC2684512

[ref61] Ramette A. (2007). Multivariate analyses in microbial ecology. FEMS Microbiol. Ecol. 62, 142–160. doi: 10.1111/j.1574-6941.2007.00375.x 17892477 PMC2121141

[ref62] Salvi P. S. Cowles R. A. (2021). Butyrate and the intestinal epithelium: modulation of proliferation and inflammation in homeostasis and disease. Cells 10:1775. doi: 10.3390/cells10071775 34359944 PMC8304699

[ref63] Singh K. M. Shah T. Deshpande S. Jakhesara S. J. Koringa P. G. Rank D. N.. (2012). High through put 16S rRNA gene-based pyrosequencing analysis of the fecal microbiota of high FCR and low FCR broiler growers. Mol. Biol. Rep. 39, 10595–10602. doi: 10.1007/s11033-012-1947-7 23053958

[ref64] Slizewska K. Markowiak-Kopec P. Zbikowski A. Szeleszczuk P. (2020). The effect of synbiotic preparations on the intestinal microbiota and her metabolism in broiler chickens. Sci. Rep. 10:4281. doi: 10.1038/s41598-020-61256-z 32152423 PMC7062770

[ref65] Tan J. McKenzie C. Potamitis M. Thorburn A. N. Mackay C. R. Macia L. (2014). The role of short-chain fatty acids in health and disease. Adv. Immunol. 121, 91–119. doi: 10.1016/B978-0-12-800100-4.00003-9 24388214

[ref66] Turner J. R. (2006). Molecular basis of epithelial barrier regulation: from basic mechanisms to clinical application. Am. J. Pathol. 169, 1901–1909. doi: 10.2353/ajpath.2006.060681 17148655 PMC1762492

[ref67] Turner J. R. (2009). Intestinal mucosal barrier function in health and disease. Nat. Rev. Immunol. 9, 799–809. doi: 10.1038/nri2653 19855405

[ref68] Umeda K. Ikenouchi J. Katahira-Tayama S. Furuse K. Sasaki H. Nakayama M.. (2006). ZO-1 and ZO-2 independently determine where claudins are polymerized in tight-junction strand formation. Cells 126, 741–754. doi: 10.1016/j.cell.2006.06.043 16923393

[ref69] Van den Abbeele P. Gerard P. Rabot S. Bruneau A. El Aidy S. Derrien M.. (2011). Arabinoxylans and inulin differentially modulate the mucosal and luminal gut microbiota and mucin-degradation in humanized rats. Environ. Microbiol. 13, 2667–2680. doi: 10.1111/j.1462-2920.2011.02533.x 21883787

[ref70] Vasai F. Brugirard Ricaud K. Bernadet M. D. Cauquil L. Bouchez O. Combes S.. (2014). Overfeeding and genetics affect the composition of intestinal microbiota in *Anas platyrhynchos* (Pekin) and *Cairina moschata* (Muscovy) ducks. FEMS Microbiol. Ecol. 87, 204–216. doi: 10.1111/1574-6941.12217 24102552

[ref71] Videnska P. Sedlar K. Lukac M. Faldynova M. Gerzova L. Cejkova D.. (2014). Succession and replacement of bacterial populations in the caecum of egg laying hens over their whole life. PloS One 9:e115142. doi: 10.1371/journal.pone.0115142 25501990 PMC4264878

[ref72] Viveros A. Chamorro S. Pizarro M. Arija I. Centeno C. Brenes A. (2011). Effects of dietary polyphenol-rich grape products on intestinal microflora and gut morphology in broiler chicks. Poult. Sci. 90, 566–578. doi: 10.3382/ps.2010-00889 21325227

[ref73] Xiao D. Shao H. Huo Y. Agung Nugroho W. Ifeoluwa Ogunniran B. Fan W.. (2022). Reclamation of ginseng residues using two-stage fermentation and evaluation of their beneficial effects as dietary feed supplements for piglets. Waste Manag. 154, 293–302. doi: 10.1016/j.wasman.2022.10.020 36308796

[ref74] Xie H. Fang J. Farag M. A. Li Z. Sun P. Shao P. (2022). Dendrobium officinale leaf polysaccharides regulation of immune response and gut microbiota composition in cyclophosphamide-treated mice. Food Chem X 13:100235. doi: 10.1016/j.fochx.2022.100235 35499019 PMC9039934

[ref75] Xie S. Z. Liu B. Ye H. Y. Li Q. M. Pan L. H. Zha X. Q.. (2019). Dendrobium huoshanense polysaccharide regionally regulates intestinal mucosal barrier function and intestinal microbiota in mice. Carbohydr. Polym. 206, 149–162. doi: 10.1016/j.carbpol.2018.11.002 30553308

[ref76] Xue L. He J. Gao N. Lu X. Li M. Wu X.. (2017). Probiotics may delay the progression of nonalcoholic fatty liver disease by restoring the gut microbiota structure and improving intestinal endotoxemia. Sci. Rep. 7:45176. doi: 10.1038/srep45176 28349964 PMC5368635

[ref77] Yang K. Lu T. Zhan L. Zhou C. Zhang N. Lei S.. (2020). Physicochemical characterization of polysaccharide from the leaf of Dendrobium officinale and effect on LPS induced damage in GES-1 cell. Int. J. Biol. Macromol. 149, 320–330. doi: 10.1016/j.ijbiomac.2020.01.026 31945440

[ref78] Ye G. Zhang L. Wang M. Chen Y. Gu S. Wang K.. (2019). The gut microbiota in women suffering from gestational diabetes mellitus with the failure of glycemic control by lifestyle modification. J. Diabetes Res. 2019:6081248. doi: 10.1155/2019/6081248 31772944 PMC6854930

[ref80] Zhang C. Chen K. K. Zhao X. H. Wang C. Geng Z. Y. (2019). Effect of l-theanine on the growth performance, immune function, and jejunum morphology and antioxidant status of ducks. Animal 13, 1145–1153. doi: 10.1017/S1751731118002884 30376911

[ref83] Zhang S. Wang H. Zhu M. J. (2019). A sensitive GC/MS detection method for analyzing microbial metabolites short chain fatty acids in fecal and serum samples. Talanta 196, 249–254. doi: 10.1016/j.talanta.2018.12.049 30683360

[ref82] Zhang X. L. Si J. P. Wu L. S. Guo Y. Y. Yu J. Wang L. H. (2013). Field experiment of F1 generation and superior families selection of Dendrobium officinale. Zhongguo Zhong Yao Za Zhi 38, 3861–3865. 24558865

[ref84] Zhang Y. Wu Z. Liu J. Zheng Z. Li Q. Wang H.. (2020). Identification of the core active structure of a Dendrobium officinale polysaccharide and its protective effect against dextran sulfate sodium-induced colitis via alleviating gut microbiota dysbiosis. Food Res. Int. 137:109641. doi: 10.1016/j.foodres.2020.109641 33233220

[ref85] Zhang Y. Zhang L. Liu J. Liang J. Si J. Wu S. (2017). Dendrobium officinale leaves as a new antioxidant source. J. Funct. Foods 37, 400–415. doi: 10.1016/j.jff.2017.08.006

[ref81] Zhang Z. Liu D. Yi B. Liao Z. Tang L. Yin D.. (2014). Taurine supplementation reduces oxidative stress and protects the liver in an iron-overload murine model. Mol. Med. Rep. 10, 2255–2262. doi: 10.3892/mmr.2014.2544 25201602 PMC4199407

[ref79] Zhan H. Q. Dong X. Y. Li L. L. Zheng Y. X. Gong Y. J. Zou X. T. (2019). Effects of dietary supplementation with *Clostridium butyricum* on laying performance, egg quality, serum parameters, and cecal microflora of laying hens in the late phase of production. Poult. Sci. 98, 896–903. doi: 10.3382/ps/pey436 30285187

[ref86] Zheng X. Xiong T. X. Zhang K. Zhou F. C. Wang H. Y. Li B.. (2021). Benefit effect of Dendrobium officinale ultrafine powder on DSS-induced ulcerative colitis rats by improving Colon mucosal barrier. Evid. Based Complement. Alternat. Med. 2021:9658638. doi: 10.1155/2021/9658638 35003313 PMC8736692

[ref87] Zhong C. Tian W. Chen H. Yang Y. Xu Y. Chen Y.. (2022). Structural characterization and immunoregulatory activity of polysaccharides from Dendrobium officinale leaves. J. Food Biochem. 46:e14023. doi: 10.1111/jfbc.14023 34873736

[ref88] Zhou G. Lv G. (2012). Comparative studies on scavenging DPPH free radicals activity of flavone C-glycosides from different parts of Dendrobium officinale. Zhongguo Zhong Yao Za Zhi 37, 1536–1540. PMID: 22993976

